# KDM4B/JMJD2B is a p53 target gene that modulates the amplitude of p53 response after DNA damage

**DOI:** 10.1093/nar/gkw1281

**Published:** 2017-01-10

**Authors:** Laura Castellini, Eui Jung Moon, Olga V. Razorenova, Adam J. Krieg, Rie von Eyben, Amato J. Giaccia

**Affiliations:** 1 Department of Radiation Oncology, Stanford University School of Medicine, Stanford, CA 94305, USA; 2 Department of Molecular Biology and Biochemistry, University of California Irvine, Irvine, CA 92697, USA; 3 Department of Obstetrics and Gynecology, Oregon Health and Sciences University, Portland, OR 97239, USA

## Abstract

The p53 tumor suppressor protein plays a critical role in orchestrating the genomic response to various stress signals by acting as a master transcriptional regulator. Differential gene activity is controlled by transcription factors but also dependent on the underlying chromatin structure, especially on covalent histone modifications. After screening different histone lysine methyltransferases and demethylases, we identified JMJD2B/KDM4B as a p53-inducible gene in response to DNA damage. p53 directly regulates *JMJD2B* gene expression by binding to a canonical p53-consensus motif in the *JMJD2B* promoter. JMJD2B induction attenuates the transcription of key p53 transcriptional targets including *p21, PIG3* and *PUMA*, and this modulation is dependent on the catalytic capacity of JMJD2B. Conversely, JMJD2B silencing led to an enhancement of the DNA-damage driven induction of *p21* and *PIG3*. These findings indicate that JMJD2B acts in an auto-regulatory loop by which p53, through JMJD2B activation, is able to influence its own transcriptional program. Functionally, exogenous expression of JMJD2B enhanced subcutaneous tumor growth of colon cancer cells in a p53-dependent manner, and genetic inhibition of JMJD2B impaired tumor growth *in vivo*. These studies provide new insights into the regulatory effect exerted by JMJD2B on tumor growth through the modulation of p53 target genes.

## INTRODUCTION

The p53 protein responds to a variety of cellular stresses, including genotoxic damage, hypoxia, nutrient depletion and aberrant proliferative signals through oncogene activation. Following stress, p53 protein translation and half-life increase, and p53 binds to DNA as a tetramer in a sequence-specific manner. The increase in p53 protein results in the transcriptional regulation of genes involved in mediating key cellular processes, such as DNA repair, cell-cycle arrest, senescence, apoptosis, autophagy and metabolism (1–3).

Modulation of chromatin through covalent histone modification is a fundamental way of regulating DNA accessibility during processes such as gene transcription, DNA replication and DNA damage repair (4). A major component in the regulation of cellular processes by chromatin structure is the post-translational modifications occurring on the N-terminal tails of histones. Such modifications include acetylation, methylation, phosphorylation, ubiquitylation and sumoylation. Each of these modifications influences the structure of chromatin and, depending on the site, the degree and the type of modification, has different functional outcomes (5). Although several histone post-translational modifications are important components of the epigenome, acetyl and methyl marks on lysine residues are the most abundant and among the most widely studied (6). Whereas lysine acetylation of histones usually correlates with transcriptional activation, histone lysine methylation can be associated with either transcriptional activation or repression depending on the residue and degree of methylation.

A plethora of histone lysine methyltransferases (HMTs) and demethylases (HDMs), which affect the dynamic regulation of lysine methylation and demethylation respectively, have been discovered in the past decade. Three families of enzymes have been identified that catalyze the addition of methyl groups to histones. The SET-domain-containing proteins and DOT1-like proteins have been shown to methylate lysines, and members of the protein arginine *N*-methyltransferase (PRMT) family have been shown to methylate arginine residues. These histone methyltransferases have the capability to methylate histones as well as non-histone proteins (7). In contrast to histone methyltransferases, two classes of histone demethylases have thus far been identified. Proteins of the KDM1 (Lysine Demethylase 1) family are FAD-dependent amine oxidases, which can act only on mono- and dimethylated lysine. Conversely, proteins containing the Jumonji C (JmjC) domain are Fe(II) and 2-oxoglutarate-dependent demethylases, which reverse mono-, di- and lysine trimethylation (8).

Although several epigenetic enzymes are able to methylate and demethylate p53 (9,10), little is known about the role of p53 in regulating these genes at the transcriptional level. We therefore wanted to elucidate whether members of the different families of methylating and demethylating enzymes might represent novel targets of p53 tumor suppressor protein. In the present study, we have screened WT and p53-deficient HCT116 colon carcinoma cells, after treatment with DNA damaging agents, for changes in the expression of genes that belong to the different families of HMTs and HDMs. Using quantitative real-time PCR (qPCR), we identified the histone demethylase 4B (KDM4B, also known as Jumonji domain-containing protein 2B, JMJD2B), as a p53 responsive gene. JMJD2B is a newly identified member of the histone demethylase JMJD2 family that is characterized by the catalytic Jumonji C (JmjC) domain. JMJD2B specifically recognizes tri- and dimethylated lysine 9 (H3K9me3/2) on histone H3, reducing both modifications to the monomethylated state (11,12). It has been recently shown that JMJD2B expression levels are notably upregulated in various cancers, including breast, colorectal, gastric, prostate, lung and bladder malignancies (13–18). Also, JMJD2B expression is controlled by the hypoxia-inducible factor 1α (HIF-1α), suggesting that JMJD2B might help tumors adapt to a hypoxic environment (19–21). After analysis of the screen, we focused on JMJD2B because of the intriguing dualism of its p53 responsiveness and its reported role in tumorigenesis. Our study shows that *JMJD2B* is induced by p53 activation, supporting the notion that it is a *bona fide* p53 target. By chromatin immunoprecipitation (ChIP) assay and functional promoter analysis, we also demonstrate that p53 can bind directly to a distal region on *JMJD2B* promoter, which has strong homology to canonical p53-binding motifs. Further, we identify a list of key p53 target genes that are affected by the overexpression of JMJD2B in the context of DNA damage response. In particular, we show that p53 was less efficient in inducing its target genes *p21, PIG3* and *PUMA* upon DNA damage under conditions in which JMJD2B expression is elevated, and most importantly JMJD2B catalytic activity is required to modulate this response. The attenuated response of key p53 targets is reflected by a decrease in the H3K4me3 permissive mark, concomitantly with an increase in the H3K9me3 repressive mark at those specific promoters. In agreement with these findings, JMJD2B silencing leads to an enhancement in the DNA-damage driven induction of the p53 targets *p21* and *PIG3*. Functionally, we show that exogenous expression of JMJD2B significantly enhances the ability of human colon cancer cells to grow *in vivo* in a p53-dependent manner, whereas genetic inhibition of JMJD2B significantly delays *in vivo* tumor growth. Taken together, these studies lead us to propose that the development of JMJD2B specific inhibitors might represent a valuable approach for cancer therapy.

## MATERIALS AND METHODS

### Cell lines, culture conditions and treatments

HCT116 p53+/+ and p53−/− colon carcinoma cells, RKO p53 wt and isogenic RKO-E6 cells transfected with a stably integrated human papillomavirus (HPV) E6 oncogene under control of the cytomegalovirus promoter, mouse embryonic fibroblasts (MEFs) and 293FT cells were maintained in Dulbecco's modified Eagle medium; HeyA8 ovarian cancer cells were grown in RPMI medium. Media were supplemented with 10% (v/v) fetal bovine serum (Omega Scientific), 2 mM L-glutamine, 100 U/ml penicillin and 100 μg/ml streptomycin. Doxorubicin, Nutlin-3, Etoposide and 5-Fluorouracil (5-FU) were purchased from Sigma-Aldrich. γ-irradiation was performed with a Mark I irradiator containing a Cesium 137 source (J.L. Sheperd and Associates).

### RNA isolation and quantitative Real-Time PCR

RNA was isolated using Trizol (Invitrogen) and subsequently treated with DNase I (Fermentas). First-strand cDNA synthesis was performed with SuperScript II Reverse Transcriptase and random primers (Invitrogen) according to the manufacturer's instructions. qPCR was carried out using Power SYBR Green Master Mix (Life Technologies), detection and data analysis were executed with the 7900HT Fast Real-Time PCR System (Applied Biosystems) by computing the results relative to a standard curve made with cDNA pooled from all samples, normalized to 18S. Primer sequences used to amplify specific target genes were obtained from the Universal Probe Library Assay Design Center (https://lifescience.roche.com/en_us/brands/universal-probe-library.html) and are listed in the Supplementary Table S3.

### Chromatin Immunoprecipitation

ChIP was performed as described previously (22), with the following modifications. Briefly, HCT116 cells were exposed to doxorubicin or 5-FU for 24 h prior to formaldehyde fixation. Fixed and lysed cells were sonicated using a Bioruptor Plus Sonication System (Diagenode) set at high power, 30 s ON, 90 s OFF, 60 cycles. Approximately 15–25 μg of sonicated chromatin was incubated overnight at 4°C with 2 μg of p53 (Santa Cruz Biotechnology), histone H3 (Abcam), histone H3K4me3 (Abcam), histone H3K9me3 (Abcam), JMJD2B (Cell Signaling) antibodies, followed by precipitation with protein A/G Dynabeads (Invitrogen). Normal mouse or rabbit IgG (Santa Cruz Biothechnology) was used as a non-specific IgG control. Approximately 5% of the sample from each immunoprecipitation was reserved for input control. Immunoprecipitated complexes were washed, eluted and reverse crosslinked. DNA was purified with QIAquick PCR purification kit following the manufacturer's protocol (Qiagen). Relative enrichment of the samples was measured by qPCR using a titration of pooled input samples as a standard curve, and normalized to input after subtraction of IgG signal. Relative occupancy is presented as percentage of input. For histone ChIPs, H3K4me3 and H3K9me3 enrichments were normalized to bulk histone H3 signal. Primer sequences are listed in Supplementary Table S3.

### shRNA, siRNA and cDNA expression constructs, lentivirus and retrovirus production and infection of target cells

Short hairpin RNA (shRNA) lentiviral constructs against human *JMJD2B* (pLKO.1-shJMJD2B) were purchased from Open Biosystems. The target sequences in *JMJD2B* mRNA are 5΄- GCCCATCATCCTGAAGAAGTA-3΄ for shRNA-2 and 5΄-GTGGAAGCTGAAATGCGTGTA-3΄ for shRNA-4. Control hairpin against GFP in the pLKO.1 backbone (pLKO.1-shGFP) was a kind gift from Silvestre Vicent (Stanford University). For lentiviral stock preparation, 293FT cells were transfected with pLKO.1-based vectors and two helper plasmids (pCMV-ΔR8.2 and pCMV-VSV-G) using Lipofectamine and Plus Reagent according to the manufacturer's protocol (Invitrogen). About 24–48 h after transfection, media containing packaged lentivirus were collected, passed through 0.45 μ filters and added to target cells along with 5 μg/ml of Polybrene (Sigma-Aldrich). Infected cells were selected in puromycin-containing media (1 μg/mL) for 1 week.

Small interfering RNA (siRNA) was carried out using Dharmacon siGENOME SMART pool siRNAs for *TP53* and *JMJD2B*, and siGENOME Non-Targeting siRNA Pool #2. Cells were transfected at a final concentration of 100 nM using DharmaFECT 1 transfection reagent, according to the manufacturer's instructions (Dharmacon).

*JMJD2B/KDM4B* full-length human cDNA was obtained from Open Biosystems (GE Dharmacon, Lafayette, CO, USA) and subcloned by HindIII and XhoI (New England Biolabs) into the retroviral expression vector pLPC (a gift from S. Lowe, Cold Spring Harbor, NY, USA) by PCR, using forward primer 5΄- AGAGAGAAGCTTAGCCATGGGGTCTGAGGACC-3΄ and reverse primer 5΄- ATATATCTCGAGGGCCAGCTGTCCTAGAAGGG-3΄. JMJD2B catalytic mutant (JMJD2B^H189A^) was generated with the QuikChange II XL site-directed mutagenesis kit (Agilent) by using forward primer 5΄-ACCACCTTCGCCTGGGCGACCGAGGACATGGAC-3΄ and reverse primer 5΄- GTCCATGTCCTCGGTCGCCCAGGCGAAGGTGGT-3΄, as per manufacturer's instructions. The JMJD2B^H189A^ construct was subcloned by HindIII and XhoI (New England Biolabs) into pLPC by PCR. All constructs were verified by sequencing. Retrovirus production and infections were done as described previously (23). Infected cells were selected in puromycin-containing medium (2 μg/ml) for 5 days.

### Reporter plasmid construction, transient transfection and luciferase assay

*JMJD2B* promoter-luciferase constructs as well as the mutant construct with altered p53-binding site were synthesized and cloned in pUC57 plasmid by GenScript (Piscataway, NJ, USA). The *JMJD2B* mutant p53-binding site #3 construct was generated by altering the CATG and CTGG p53-consensus half-sites in TCCC and TCCC, respectively. DNA regions containing BS3, mut BS3 or BS6/7 sites were subsequently inserted by NheI and XhoI (New England Biolabs) into the pGL3-Promoter vector upstream of the firefly luciferase gene (Promega), while the insert containing BS9 and BS10 sites was subcloned by NheI and HindIII into the pGL3-Basic vector. All constructs were verified by sequencing. Transient transfection of luciferase-reporter plasmids was mediated by Lipofectamine and PLUS Reagent according to the manufacturer's instructions (Invitrogen). Twenty-four hours after transfection, HCT116 p53+/+ and p53−/− cells were exposed to doxorubicin for 18 h or left untreated. Alternatively, HCT116 p53−/− cells were co-transfected with either pLPC-empty vector (pLPC(EV)) or pLPC vector carrying human N-FLAG-p53 (pLPC-p53) along with the luciferase-reporter plasmids BS3 or mut BS3, following doxorubicin treatment. Cells were lysed and assayed for firefly luciferase activity using a Bright-Glo Luciferase Assay System (Promega) on a Monolight 2010 luminometer (Analytical Luminescence Laboratory). Assays were performed in duplicate and repeated three times.

### Protein isolation and Western blotting

For protein analysis, cells were harvested in lysis buffer (20 mM Tris-HCl pH 7.5, 150 mM NaCl, 1 mM EDTA pH 8.0, 1 mM EGTA pH 8.0, 1% Triton X-100) supplemented with protease and phosphatase inhibitor cocktails (Roche), then incubated on ice for 15 min, vortexed and centrifuged at 13 000 rpm. Protein lysates were quantified using BCA Protein Assay kit (Pierce) and 25–100 μg of protein samples were resolved by SDS-PAGE according to standard methods, then transferred onto 0.2 μm Supported Nitrocellulose membranes (Bio-Rad Laboratories). The following primary antibodies were used to detect specific proteins: JMJD2B (#2898 and #8639, Cell Signaling), p53 (DO-1, Santa Cruz Biotechnology), phospho-p53 S15 (Cell Signaling), p21 (Santa Cruz Biotechnology), PIG3 (Oncogene Research Products), MDM2 (Santa Cruz Biotechnology), histone H3K9me3 (Active Motif), Hsp70 (Sigma-Aldrich), α-tubulin (Fitzgerald Industries International), β-actin (Sigma-Aldrich). Secondary antibodies used in this study were HRP-conjugated goat anti-mouse and anti-rabbit (Vector Laboratories). Immunoblots were developed with SuperSignal West Dura Extended Duration Substrate (Thermo Fisher Scientific) and visualized with ChemiDoc XRS+ imaging system equipped with Image Lab Software (Bio-Rad Laboratories). Protein bands were quantified by densitometry using ImageJ software (National Institutes of Health, Bethesda, MD, USA).

### Immunoprecipitation

Protein lysates were prepared and quantified as described under Western blotting section. Approximately 250 µg of proteins were pre-incubated with Protein A/G Dynabeads (Thermo Fisher Scientific), followed by overnight incubation with 2 μg of p53 DO-1 antibody (Santa Cruz Biotechnology) at 4°C with gentle agitation. Normal mouse IgG (Santa Cruz Biothechnology) was used as a non-specific IgG control, and 10% of the sample from each immunoprecipitation was reserved for input control. Subsequently, lysates were incubated with 25 μl of Protein A/G Dynabeads for 3 h at 4°C and immunoprecipitated proteins were collected using magnetic stand, washed three times, boiled in 1× sample loading buffer for 10 min and analyzed by WB.

### Immunohistochemistry

For xenograft studies, tumor specimens were fixed in 10% (v/v) neutral buffered formalin, permeabilized with 95% (v/v) ethanol and embedded in paraffin. Tumor sections were subsequently deparaffinized with xylene, rehydrated in ethanol solutions and subjected to antigen retrieval using 10 mM citric acid buffer (pH 6.0) in microwave for 10 min. Slides were probed with primary anti-Ki67 antibody (Thermo Fisher Scientific) overnight at 4°C, followed by secondary detection using biotinylated anti-rabbit antibody (Vector Laboratories) and streptavidin-HRP conjugated antibody (EMD Millipore), both for 30 min at 37°C. Negative controls for all samples were tissue sections treated with secondary antibodies alone. Proteins were visualized with DAB Chromogen System (DAKO), counterstained with Hematoxylin (VWR) and mounted on slides with Fluoromont-G (Southern Biotech). Pictures were captured using a Leica DM6000B microscope (Leica Microsystems) equipped with Image-Pro Premiere 9.0 software (Media Cybernetics).

### Tumor xenografts

All procedures involving animals and their care were performed in accordance with Institutional and National guidelines, and approved by Stanford University's Administrative Panel on Laboratory Care (APLAC). HCT116 p53+/+ and p53−/− cells stably expressing pLPC(EV) and pLPC-JMJD2B, or carrying shGFP, shJMJD2B-2 and shJMJD2B-4 lentiviral constructs were implanted subcutaneously into the lower flanks of SCID Hairless 6–8 week-old female mice (Charles River Laboratories). Tumors were measured with calipers at regular intervals, and tumor volume was calculated according to the following formula: volume = (width^2^ × length) × 0.5.

### Statistics

All statistical analyses were generated using Prism software (Graphpad). Significance was determined by two-tailed Student's *t*-test. Alternatively, data were analyzed in an ANOVA model and pairwise comparisons were done with a Tukey adjustment. For all analyses *P* < 0.05 was considered statistically significant, and *P < 0.05, **P < 0.01, ***P < 0.001, ****P < 0.0001.

## RESULTS

### Differential regulation exerted by p53 on the expression of a panel of histone modifying enzymes

**Figure 1.**
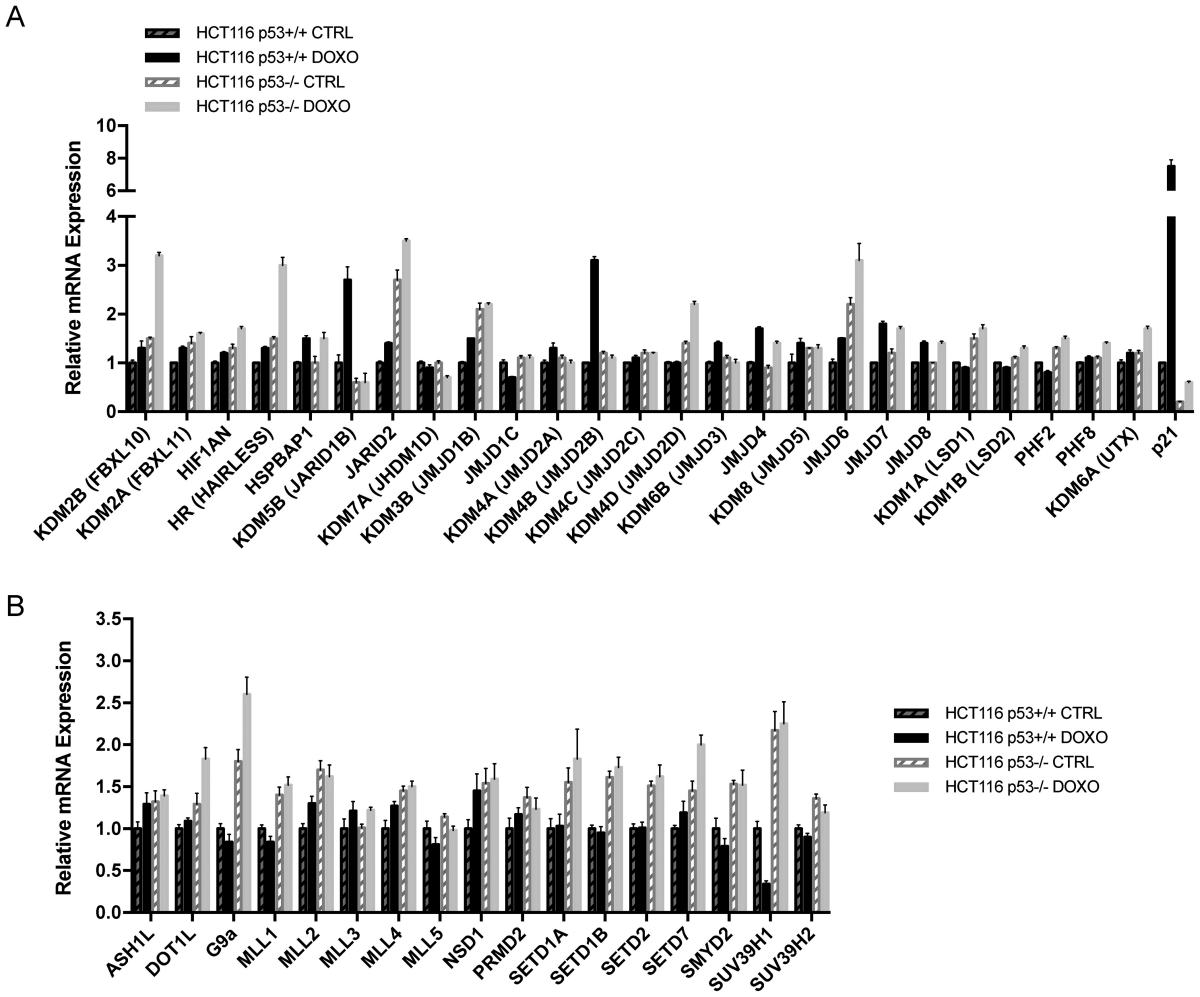
HDMs and HMTs screen reveals differential regulation exerted by p53 following DNA damage. (**A** and **B**) qPCR showing mRNA expression of the indicated HDMs (A) and HMTs (B) in p53+/+ and p53−/− HCT116 colon carcinoma cell lines treated with 0.3 μg/ml doxorubicin (DOXO) for 24 h or left untreated (CTRL). *p21* serves as control for p53-specific gene. Data represent the averages from three independent experiments, measured in triplicate and normalized to 18S rRNA. Data are presented as fold change relative to HCT116 p53+/+ CTRL cells. Error bars indicate standard error of the means (±SEM). See also Supplementary Figure S1 and Supplementary Table S1.

In order to identify novel p53-transcriptionally regulated genes among the different families of epigenetic enzymes, we performed a qPCR screen by analyzing changes in the expression of HDMs and HMTs in cells harboring a WT *TP53* gene (HCT116 p53+/+ cells) and in the isogenic cell line lacking *TP53* (HCT116 p53−/−), following treatment with the chemotherapic agent doxorubicin. The screening of 25 proteins comprising both families of amine oxidase and jumonji C–domain containing iron-dependent dioxygenases, several of which have been found to possess histone demethylase activity, revealed different patterns of regulation following DNA damage (Figure 1A). In particular, a subset of genes, including the histone demethylase *JMJD2B/KDM4B* and the lysine-specific demethylase 5B (*KDM5B*, also named jumonji, AT rich interactive domain 1B, *JARID1B*), was found to be regulated by p53. These genes were induced about 2.5-fold selectively in HCT116 p53+/+ cells, but not in HCT116 p53−/− cells in response to doxorubicin treatment. A second group of genes, including *JARID2* (jumonji, AT rich interactive domain 2 demethylase), *JMJD6* (jumonji domain containing 6 demethylase) and the lysine-specific demethylase 1A (*KDM1A*, also named lysine-specific demethylase 1, *LSD1*), was selectively repressed by p53. Indeed, their transcript levels were increased about 1.5–2-fold in both control and doxorubicin-treated HCT116 p53−/− cells. Finally, a third subset of genes, including the hair growth associated gene HR (also known as HAIRLESS), the lysine-specific demethylase 2B (KDM2B, also known as F-box protein 10, FBXL10) and the lysine-specific demethylase 4D (KDM4D, also named jumonji domain-containing protein 2D, JMJD2D), was induced in response to the DNA damaging agent, but in a p53-independent manner. In fact, only HCT116 cells lacking p53 protein showed a 2–2.5-fold increase in the expression of these epigenetic enzymes in response to doxorubicin treatment, and their mRNA levels were unchanged in unstressed conditions.

Among the first group of genes identified by this screen, *JMJD2B* was of particular interest given the apparent paradox of being activated by p53, yet being recently reported to be overexpressed in numerous cancers (24,25) and involved in the disruption of pericentromeric heterochromatin that promotes chromosome instability (26).

In contrast, the screening of 17 histone methyltransferases (Figure 1B), comprising both families of SET-domain-containing proteins and DOT1-like proteins, mainly revealed one pattern of regulation. Genes including *KMT2A* (lysine-specific methyltransferase 2A, also named mixed lineage leukemia 1, *MLL1*), *KMT2B* (lysine-specific methyltransferase 2B, also named mixed lineage leukemia 2, *MLL2*), *SETD1B* (SET domain containing 1B), *SETD1A* (SET domain containing 1A), *SETD7* (SET domain containing-lysine methyltransferase 7), *SETD2* (SET domain containing-lysine methyltransferase 2), *EHMT2* (euchromatic histone-lysine N-methyltransferase 2, also named *G9A*), *SMYD2* (SET and MYND domain containing 2) and *SUV39H1* (suppressor of variegation 3–9 homolog 1 (Drosophila)) displayed a marked repression exerted by p53. Indeed, their transcript levels were increased about 1.5-fold in both control and doxorubicin-treated HCT116 p53−/− cells. This result is in line with the recent findings of Mungamuri *et al.* and Zheng *et al.*, showing the ability of p53 to influence its own transcriptional program by downregulating the expression of the histone methyltransferase SUV39H1 (27,28).

### *JMJD2B* expression is induced in a p53-dependent manner

**Figure 2.**
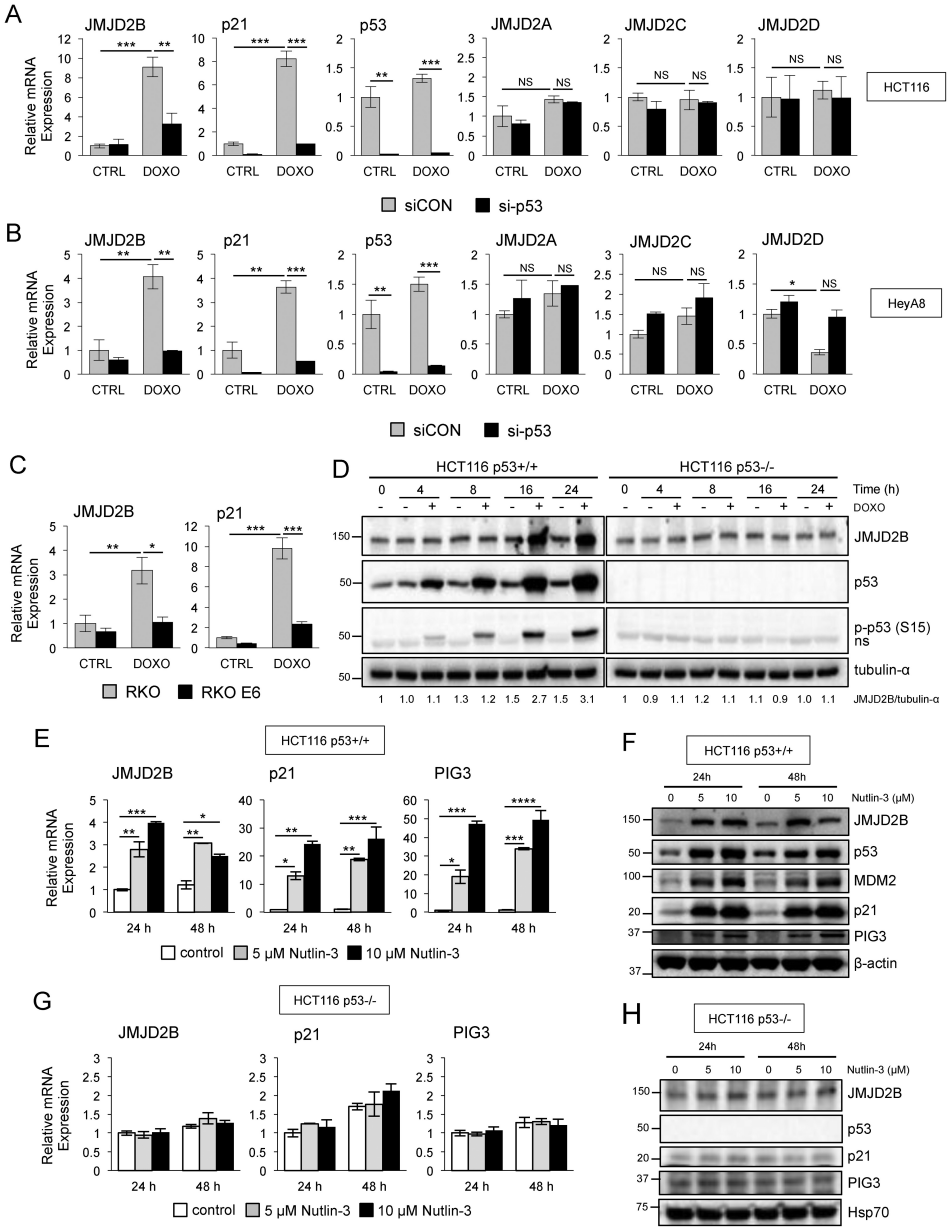
p53-dependent regulation of JMJD2B in response to doxorubicin and Nutlin-3 agents. (**A** and **B**) QPCR analysis of *JMJD2A, JMJD2B, JMJD2C, JMJD2D, p21* and *p53* mRNA expression in HCT116 (A) and HeyA8 (B) cells transiently transfected with siRNAs targeting p53 (si-p53) or with a non-targeting control siRNA (siCON), and treated with 0.3 μg/ml doxorubicin for 24 h (*n* = 3 per group). Values measured in triplicate, normalized to 18S, ±SEM and presented as fold change relative to the untreated (CTRL) siCON cells. ^∗^*P* < 0.05; ^∗∗^*P* < 0.01; ^∗∗∗^*P* < 0.001; NS, *P* > 0.05 (ANOVA). *p53* serves as control for the efficacy of the siRNA treatment. See also Supplementary Figure S2. (**C**) QPCR analysis of *JMJD2B* and *p21* mRNA expression in RKO and RKO-E6 cells following 24 h of doxorubicin treatment. (**D**) Lysates from p53+/+ and p53−/− HCT116 cells treated with 0.3 μg/ml of doxorubicin were prepared at the indicated time points and analyzed by western blotting using anti-JMJD2B, anti-p53 and anti-phospho-p53 (p-p53 (S15)) antibodies. Densitometry (ImageJ software) is shown with tubulin-α used as protein loading control; ‘ns’ represents a nonspecific band. (**E** and **F**) p53 stabilization by Nutlin-3 results in JMJD2B induction in HCT116 p53+/+ cells but not in HCT116 p53−/− cells (**G** and **H**). QPCR (E–G) and Western blot analysis (F–H) of HCT116 p53+/+ and HCT116 p53−/− cells, respectively, treated with 5 or 10 μM Nutlin-3 for the indicated time points showing JMJD2B expression. p21 and PIG3 were used as positive controls. Values measured in triplicate, normalized to 18S, ±SEM and presented as fold change relative to control cells at 24 h. ^∗^*P* < 0.05; ^∗∗^*P* < 0.01; ^∗∗∗^*P* < 0.001; ^∗∗∗∗^*P* < 0.0001 (ANOVA). β-actin and Heat shock protein 70 (Hsp70) used as protein loading control.

To further confirm the results of the screen and to determine whether regulation of *JMJD2B* by p53 also occurred in different cancer cell lines, we analyzed the expression of *JMJD2B* by qPCR in cells transiently transfected with siRNA duplexes targeting p53 (si-p53) or a non-targeting control siRNA (siCON), and treated with doxorubicin. Consistent patterns of *JMJD2B* induction upon DNA damage were observed in the two cell lines treated with a non-targeting control siRNA. Specifically, HCT116 cells exhibited about 9-fold induction (Figure 2A) and the ovarian cancer cell line HeyA8 showed about 4-fold induction (Figure 2B) of *JMJD2B* transcripts. In contrast, specific inhibition of p53 expression reduced the induction of *JMJD2B* in response to doxorubicin in both HCT116 and HeyA8 cells in a statistically significant manner. Efficacy of siRNA delivery was tested, and p53 gene silencing ranged from 90 to 95% in treated and untreated samples. DNA-damage driven induction of *p21* mRNA was also reduced in a statistically significant manner by knocking down p53.

The JMJD2/KDM4 family is composed of four members, JMJD2A, JMJD2B, JMJD2C and JMJD2D. The first three members encompass the catalytic JmjC domain, the JmjN domain, two PHD and two Tudor domains. In contrast, JMJD2D is the most structurally divergent JMJD2 protein as it lacks the PHD and Tudor domains (12,29). Recent studies indicate that JMJD2B is regulated by hypoxia, but other JMJD2B/KDM4 family members do not robustly respond to changes in oxygen levels (19–21). These findings prompted us to investigate whether the regulation exerted by p53 was specific to JMJD2B or extended to the other members of the JMJD2 family. Quantitative Real-Time PCR analysis confirmed that *JMJD2B* mRNA, but not *JMJD2A, JMJD2C* or *JMJD2D* mRNAs, was induced upon doxorubicin treatment, in both HCT116 (Figure 2A) and HeyA8 (Figure 2B) cells. In addition, targeting p53 expression by means of siRNA transfection specifically reduced the induction of *JMJD2B* in response to doxorubicin, while it did not affect the expression of the three other family members, clearly demonstrating the specificity of *JMJD2B* upregulation in response to p53 activation. Similarly, *JARID1B* mRNA levels were increased after doxorubicin treatment in both HCT116 (Supplementary Figure S1A) and HeyA8 (Supplementary Figure S1B) cells in a p53-dependent manner, whereas *JARID1A* and *JARID1C* had no dependence on p53 for expression.

Interestingly, kinetic analysis of *JMJD2B* expression in response to DNA damage revealed that, in HCT116 p53+/+ cells, transcriptional induction of *JMJD2B* started 10 h after exposure to doxorubicin and increased 6-fold compared to the untreated control at 24 h (Supplementary Figure S2). Similar results were observed in HeyA8 cells. Although the amplitude and kinetics of activation differed between the genes and cells screened, the concordance between sustained activation of the two p53 targets, *p21* and *PCNA*, with the expression of *JMJD2B* is consistent with the recognition of JMJD2B as an effector of the p53 pathway. In contrast to *JMJD2B*, we found that *JMJD1A* had no dependence on p53 for expression after doxorubicin treatment in both HCT116 and HeyA8 cells, highlighting the specificity of *JMJD2B* regulation in response to p53 activation (Supplementary Figure S2).

*JMJD2B* expression was also induced in RKO colon carcinoma cells, carrying WT p53 alleles, upon DNA damage treatment, showing a 3.1-fold increase compared to the untreated control (Figure 2C). In contrast, an isogenic cell line genetically engineered to express the HPV E6 oncoprotein (RKO-E6), which binds to WT p53 protein (30,31) and mediates its degradation *in vitro* through an ubiquitin-dependent mechanism (32), did not show any significant increase in *JMJD2B* mRNA after doxorubicin treatment compared to the untreated controls. The known p53 transcriptional target *p21* served as positive control (33).

To determine if changes in *JMJD2B* mRNA were mirrored by changes at the protein levels, we monitored JMJD2B protein induction after exposure to doxorubicin in both p53+/+ and p53−/− HCT116 cells (Figure 2D). Doxorubicin induced a significant increase of JMJD2B protein in WT p53 cells, which was associated with phosphorylation of p53 on serine 15, but had no effect on HCT116 cells lacking p53.

Since the expression of *JMJD2B* was induced by p53 activation in response to doxorubicin treatment, we expected that p53 stabilization/accumulation, in the absence of DNA damage, could also elevate the expression of *JMJD2B* mRNA in the same cells. To test our hypothesis, we used Nutlin-3, a small-molecule known to be a MDM2 antagonist. Nutlin-3 specifically binds to MDM2 and disrupts MDM2-p53 interaction, resulting in a dramatic stabilization of p53 and activation of the p53 pathway (34). Following Nutlin-3 treatment, JMJD2B expression, at both mRNA (Figure 2E) and protein (Figure 2F) levels, was significantly increased in HCT116 cells, in a dose-dependent manner, as was that of p53 targets p21 and PIG3. Similarly, we found that also *JARID1B* mRNA levels were increased after Nutlin-3 treatment in HCT116 cells (Supplementary Figure S1C). Importantly, JMJD2B induction was not observed in HCT116 p53−/− cells exposed to Nutlin-3, at both mRNA (Figure 2G) and protein levels (Figure 2H). Together, these experiments clearly demonstrate that *JMJD2B* is induced upon p53 activation, in different cancer cell lines, either upon DNA damage or increased p53 stabilization by Nutlin-3, supporting the concept that *JMJD2B* is a *bona fide* p53 target. In addition, targeting *p53* expression by means of siRNA transfection specifically reduced the induction of *JMJD2B* in response to doxorubicin, while it did not affect the expression of the three other family members, supporting the specificity of JMJD2B upregulation in response to p53 activation.

### *JMJD2B* is induced in response to DNA damaging agents

**Figure 3.**
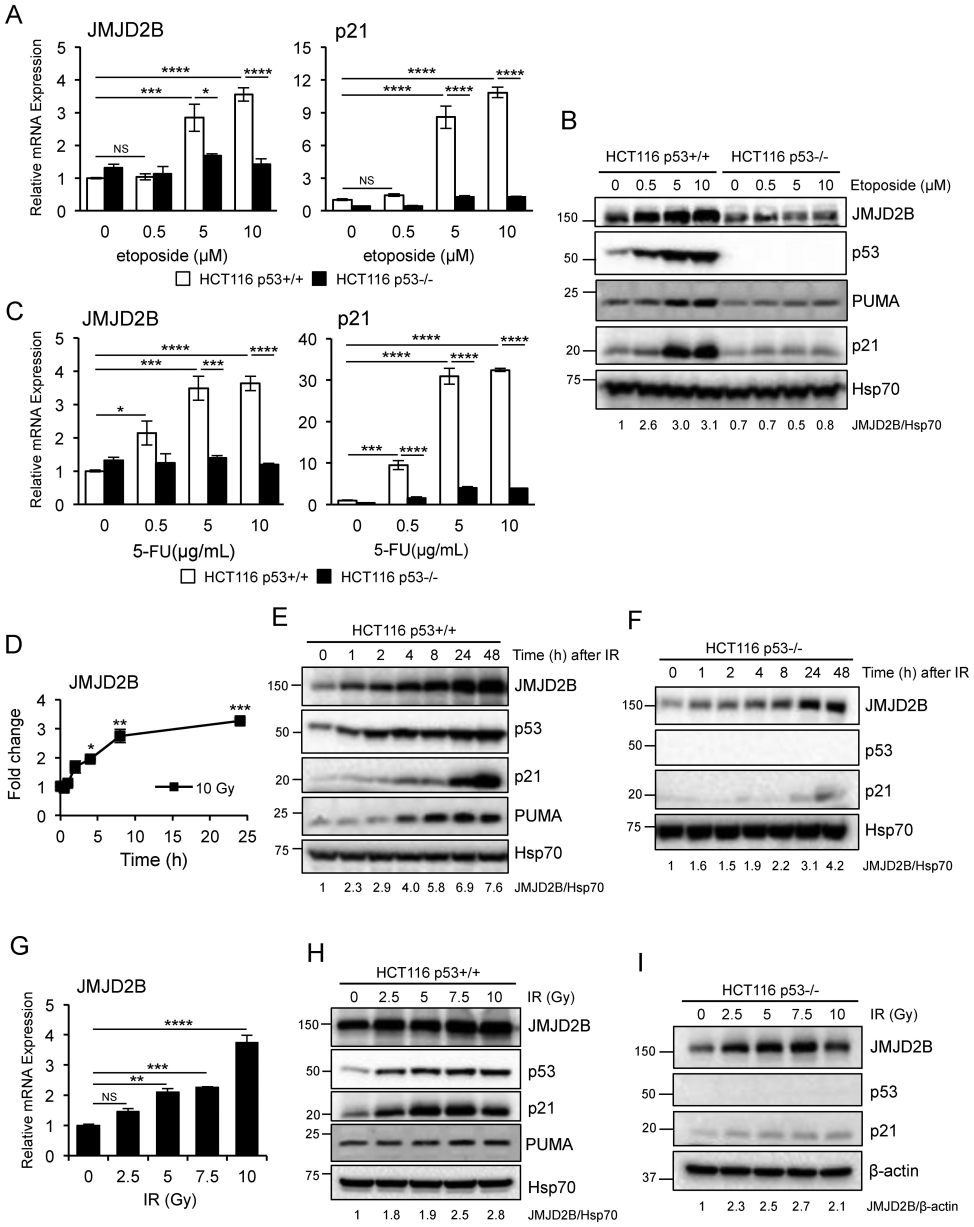
JMJD2B is induced by p53 after exposure to different DNA damaging agents. (**A** and **B**) QPCR analysis (A) and Western blotting showing mRNA levels (A) and protein expression (B) of JMJD2B and p21 in HCT116 p53+/+ and p53−/− cells treated with etoposide, at the indicated doses, for 24 h (n = 2 per group). (**C**) QPCR of *JMJD2B* and *p21* expression in HCT116 p53+/+ and p53−/− cells treated with 5-fluorouracil (5-FU). (D–F) QPCR (**D**) and Western blot analysis (**E** and **F**) showing that γ irradiation results in JMJD2B induction in HCT116 WT as well as in p53 null cells. Cells were exposed to 10 Gy, and RNAs and protein lysates were collected 0.5, 1, 2, 4, 8, 24 h later. Data plotted as fold change expression versus the untreated sample. (**G–I**) Cells were exposed to the indicated doses of ionizing radiation, and RNAs (G) and protein lysates from HCT116 WT (H) as well from p53 KO (I) cells were collected 24 h later. All values measured in triplicate, normalized to 18S and presented as fold change relative to control cells, ±SEM. ^∗^*P* < 0.05; ^∗∗^*P* < 0.01; ^∗∗∗^*P* < 0.001; ^∗∗∗∗^*P* < 0.0001; NS, *P* > 0.05 (ANOVA). Densitometry is shown with Hsp70 or β-actin used as protein loading controls.

To assess how broadly *JMJD2B* was induced by p53 in response to DNA damage, we examined its activation in response to additional genotoxic agents, such as etoposide, 5-fluorouracil (5-FU) and ionizing radiation (IR). We found that JMJD2B expression, at both mRNA (Figure 3A) and protein (Figure 3B) levels, was significantly induced after a treatment with 5 or 10 μM etoposide, in HCT116 p53+/+ cells, but not in HCT116 p53−/− cells. The same effect was observed for the p53 targets p21 and PUMA. Moreover, *JMJD2B* mRNA expression gradually increased upon 5-FU treatment in a dose dependent manner in HCT116 p53+/+ cells, but not in HCT116 lacking p53 expression, as was that of *p21* (Figure 3C). Additionally, *JMJD2B* transcript was significantly upregulated in WT HCT116 cells exposed to 10 Gy IR after a 24 h time course analysis (Figure 3D), with JMJD2B protein showing similar kinetics (Figure 3E). JMJD2B mRNA (Figure 3G) and protein (Figure 3H) expression also increased in a dose dependent manner in the same cell line treated with different IR doses. In contrast to etoposide and 5-FU treated cells, HCT116 p53−/− cells showed a significant increased in JMJD2B protein levels following IR exposure (Figure 3F and I), indicating that JMJD2B responsiveness to IR is mediated by both p53-dependent and p53-independent pathways.

### *JMJD2B* is a direct p53 target gene

**Figure 4.**
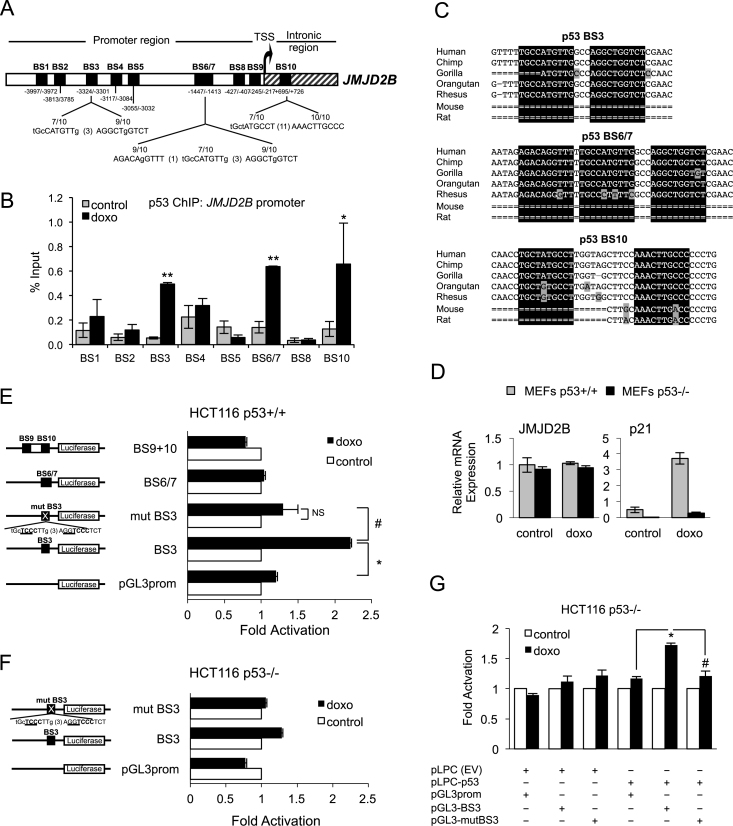
The *JMJD2B* promoter contains functional p53 binding elements. (**A**) Schematic representation of the human *JMJD2B* promoter spanning 5 kb upstream and 1 kb downstream of the transcriptional start site (TSS). Ten potential p53 binding sites (BS) are indicated with black boxes. BS3, BS6/7 and BS10 are compared to the p53-canonical consensus sequence (see also Supplementary Table S2). (**B**) ChIP assay showing p53 recruitment to the *JMJD2B* promoter. HCT116 cells were treated with 0.3 μg/ml doxo or left untreated (control) for 24 h, fixed in formaldehyde and interrogated by ChIP with antibodies against p53 and normal rabbit IgG. The precipitated DNA was amplified by qPCR using specific primers flanking the p53 BS depicted in (A). See also Supplementary Figure S3 and Supplementary Table S3. Enrichments were measured using a titration of pooled input samples as a standard curve, and are presented as percentage of input, ± SEM. ^∗^*P* < 0.05; ^∗∗^*P* < 0.01 (Student's *t*-test). (**C**) Genomic DNA alignment of regions containing BS3, BS6/7 and BS10 in *JMJD2B* gene promoter among different mammalian species. Analysis carried out using the UCSC Genome Browser, Human Feb. 2009 (GRCh37/hg19) Assembly. Boxes highlighted in black refer to half sites of the consensus p53 BS. Gray boxes refer to mismatches with respect to the human sequence. (**D**) QPCR analysis of murine *JMJD2B* expression in WT and p53−/− MEFs following 24 h treatment with doxo. (**E** and **F**) Reporter assay showing luciferase induction in HCT116 p53+/+ (E) and p53−/− (F) cells transiently transfected with constructs carrying the indicated p53 BS on *JMJD2B* promoter, cloned into a pGL3-Promoter vector upstream of the firefly luciferase gene, as well as construct with mutated p53 BS3, upon treatment with 0.5 μg/ml doxo for 24 h. Schematic representation of the JMJD2B luciferase reporter constructs is shown on the left panels. Alteration of p53-binding site 3 (mutBS3) is shown with mutations underlined. (**G**) HCT116 p53−/− cells were co-transfected with either an empty vector (pLPC(EV)) or pLPC vector carrying human N-FLAG-p53 (pLPC-p53) along with either luciferase-construct containing p53 BS3 or a mutated version (mut BS3). Cells were subsequently treated with 0.5μg/ml doxo for 24 h, and luciferase activity was assessed. In E through G, luciferase activity in control samples was designated as 1 and data represent the mean relative fold induction of luciferase activity ±SEM of three independent replicates. ∗ and #, *P* < 0.05 (Student's *t*-test). NS, *P* > 0.05.

Since our data indicate that *JMJD2B* is a p53-responsive gene, and p53 primarily functions as a transcription factor that binds to target DNA sequences, we wanted to establish whether *JMJD2B* is a direct transcriptional target of p53. We therefore scanned the human *JMJD2B* genomic region spanning 5 kb upstream and 1 kb downstream of the TSS for potential p53-recognition sequences. Ten putative p53-binding sites (BS) were identified, according to the consensus sequence RRRC(A/TA/T)GYYY (N)_0-13_ RRRC(A/TA/T)GYYY (35), within the *JMJD2B* promoter and the first intron of the gene (Figure 4A and Supplementary Table S2). p53 ChIP was performed with nuclear extracts from HCT116 p53+/+ cells exposed to doxorubicin (Figure 4B). Endogenous p53 was found to bind to an intronic site (BS10), as well as to proximal (BS 6/7) and distal regions (BS3) of *JMJD2B* promoter, with the most substantial enrichment being at −3324/−3301 bp (BS3) from the TSS (over 9-fold enrichment upon DNA damage treatment compared to control). The occupancy of p53 on the *PCNA* promoter, an established p53 target gene (36), was used as positive control (Supplementary Figure S3). Interestingly, when we compared the human p53-response elements and flanking nucleotides located on the *JMJD2B* genomic sequence identified in our study with those of other mammalian species, the genomic alignment revealed that p53-binding sites BS3, BS6/7 and BS10 were highly conserved in primates but absent in mouse or rat (Figure 4C). Notably, we were unable to detect any significant increase in *JMJD2B* mRNA levels in WT as well as p53−/− MEFs treated with doxorubicin (Figure 4D), indicating that transactivation of *JMJD2B* by p53 is not conserved between mice and humans. ChIP analysis performed on *JARID1B* promoter also revealed two regions of p53 enrichment in response to doxorubicin located approximately 3.6 and 4 kb upstream of the transcription start site (BS4 and BS5) (Supplementary Figure S1D).

To further evaluate whether p53 can directly activate transcription of *JMJD2B* and to pinpoint the functional p53-consensus, we generated several reporter constructs by subcloning promoter fragments containing BS3, BS6/7 or BS9+10 into the pGL3-Promoter luciferase reporter vector (Figure 4E). We also generated another construct, mut BS3, in which BS3 was mutated at key p53 consensus nucleotides (CATG to TCCC in the first half-site, CTGG to TCCC in the second half-site). To determine the responsiveness of *JMJD2B* regulatory regions to endogenous p53, HCT116 WT cells were transfected with the different reporter constructs and luciferase activity was measured upon doxorubicin treatment. We found that luciferase activity in cells harboring BS3 was significantly increased (2.2-fold induction) in response to doxorubicin relative to the vector control (pGL3prom), but not in cells transfected with BS6/7 or BS9+10. Furthermore, mutation of the BS3 site completely abolished this response, demonstrating that the observed p53-dependent activity is mediated through this consensus motif. In order to confirm that the *JMJD2B* regulatory region BS3 responds specifically to p53, the same reporter assay was conducted using HCT116 p53−/− cells (Figure 4F). We observed no significant changes in luciferase activity when p53-deficient cells were transfected with BS3 construct compared to the vector control upon DNA damage treatment, as well as no difference in cells carrying mutated BS3 construct. We also employed the converse approach by introducing exogenous WT human p53 into HCT116 p53-deficient cells. Luciferase activity of cells co-transfected with pLPC vector carrying human N-FLAG-p53 (pLPC-p53) and the BS3 reporter construct was enhanced 1.7-fold relative to the vector control in response to doxorubicin, whereas mutant BS3 construct failed to respond to exogenous p53 (Figure 4G). Collectively, these results clearly demonstrate that *JMJD2B* is a direct transcriptional target of p53 in human cells, and functional p53 is necessary for *JMJD2B* induction in response to genotoxic stress.

### JMJD2B overexpression attenuates the response to genotoxic stress of selected p53 target genes

**Figure 5.**
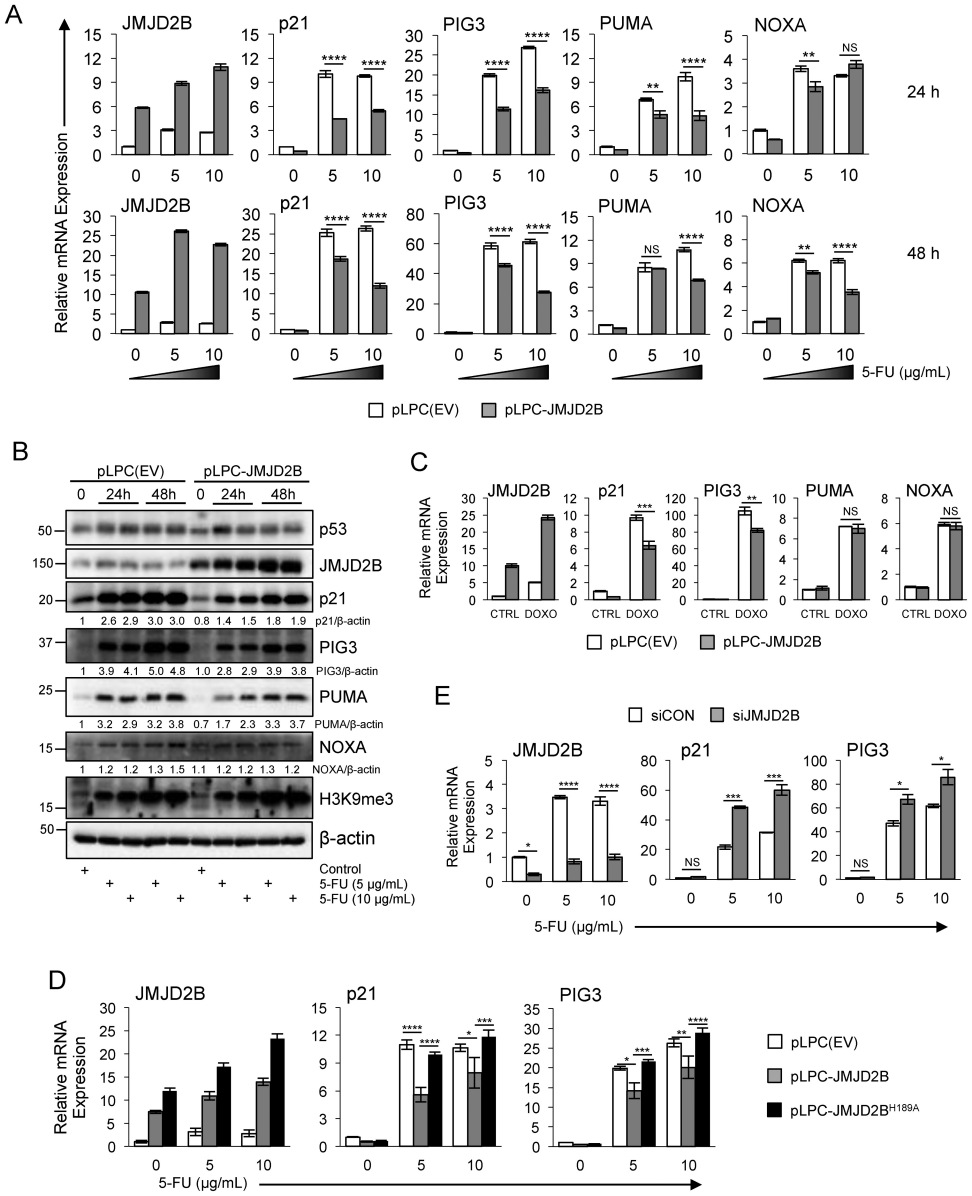
Reduced ability of p53 to transactivate a subset of its target genes when JMJD2B is overexpressed. (**A**) HCT116 p53 WT cells, transfected with pLPC-empty vector or pLPC-JMJD2B retroviral construct, were treated with 5 or 10 μg/ml of 5-FU, or left untreated. Total RNA was collected 24 or 48 h later, and expression levels of the indicated transcripts were determined by qPCR analysis. Values measured in triplicate and normalized to 18S, ±SEM. ^∗∗^*P* < 0.01; ^∗∗∗∗^*P* < 0.0001; NS, *P* > 0.05 (ANOVA). (**B**) Western blot analysis of HCT116 p53+/+ cells carrying pLPC-empty vector or pLPC-JMJD2B, treated with 5 or 10 μg/ml of 5-FU as in (A). Cell extracts were resolved by SDS-PAGE and subjected to immunoblotting with the indicated antibodies. β-actin was used as protein loading control. (**C**) QPCR analysis showing mRNA levels of *JMJD2B, p21, PIG3, PUMA* and *NOXA* in HCT116 p53 WT cells overexpressing JMJD2B or control cells (pLPC(EV)), following treatment with 0.3 μg/ml doxorubicin. Values measured in triplicate and normalized to 18S, ±SEM. (**D**) Inactivation of the catalytic function of JMJD2B (H189A mutation in the JmjC domain) resulted in a complete abrogation of the attenuate response of *p21* and *PIG3* expression observed with ectopic expression of full-length WT JMJD2B. Graphs represent qPCR analysis of *JMJD2B, p21* and *PIG3* transcripts in HCT116 p53+/+ cells stably infected with the indicated constructs upon 5-FU treatment (*n* = 4; errors bars ± SEM; ANOVA). (**E**) *JMJD2B* silencing caused an enhancement in the DNA-damage driven induction of the p53 targets *p21* and *PIG3*. QPCR measurements of the indicated transcripts in HCT116 p53 WT cells transiently transfected with siRNAs targeting JMJD2B (siJMJD2B) or with a non-targeting control siRNA (siCON), and treated with 5-FU for 24 h (*n* = 2 per group). Error bars ± SEM. ^∗^*P* < 0.05; ^∗∗^*P* < 0.01; ^∗∗∗^*P* < 0.001; ^∗∗∗∗^*P* < 0.0001; NS, *P* > 0.05 (ANOVA).

The p53 transcription factor regulates the expression of an array of different genes, which mediate the p53 response to different forms of stress (1). Activation of p53 can provoke diverse cellular outcomes in response to DNA damage, the classical ones being apoptosis, senescence and cell cycle-arrest. The first two responses are terminal for the cell, whereas cell cycle-arrest permits repair processes to take place and the potential to reverse DNA damage (37). The choice between these three responses in a stressed cell depends on different variables, but it is likely that unique sets of p53-regulated genes are fine tuned in these transcriptional responses. Thus, we wanted to identify a list of key p53 target genes that were modulated by the expression of JMJD2B in response to DNA damage. The effect of JMJD2B upregulation on p53 transcriptional activity was determined by infecting HCT116 p53+/+ cells with pLPC-JMJD2B retroviral construct or pLPC-empty vector as a control (Figure 5A). HCT116 WT cells stably expressing JMJD2B showed about 5.8-fold induction of *JMJD2B* transcripts with respect to the untreated control cells. Following DNA damage by 5-FU administration for 24 h, *JMJD2B* mRNA levels increased about 3-fold versus the untreated samples for pLPC(EV)-transfected cells, and about 1.5-fold versus the control samples for pLPC-JMJD2B overexpressing cells. These effects were even more profound in cells challenged with 5-FU for 48 h. We found that *p21* transcripts were induced following DNA damage in cells treated with either 5 or 10 μg/ml of 5-FU at both 24 and 48 h time points, and this induction was significantly diminished by the overexpression of JMJD2B. Similarly, the mRNA levels of *PIG3* (p53 inducible protein 3, also known as Tp53i3) were increased more than 20-fold versus the untreated samples for pLPC(EV)-transfected cells and about 15-fold versus the unstressed samples for pLPC-JMJD2B overexpressing cells, but overall *PIG3* mRNA induction was robustly attenuated by JMJD2B following DNA damage (Figure 5A). We also observed a reduced ability of p53 to transactivate its target gene *PUMA* (p53-upregulated modulator of apoptosis), another key modulator of p53-mediated apoptotic activity, in the context of JMJD2B overexpression. In fact, *PUMA* transcript levels were reduced in cells expressing pLPC-JMJD2B versus pLPC(EV) transfected cells, with a rather modest effect when cells were treated with a lower dose of 5-FU, but with a more profound effect in cells challenged with 10 μg/ml of 5-FU. Whereas the expression of *p21, PIG3* and *PUMA* was significantly impaired after JMJD2B overexpression at both time points, *NOXA* transcript levels were not affected at 24 h but modestly, although significantly, reduced at 48 h post stimulation with 5-FU.

In line with these results, we observed a significant reduction of p21, PIG3 and PUMA proteins accumulation in cells expressing pLPC-JMJD2B compared to control cells following DNA damage, at both 24 and 48 h time points, with a more robust effect after a 48 h treatment (Figure 5B). Also, NOXA protein levels were not affected at 24 h while moderately reduced after cells were exposed to 10 μg/ml of 5-FU for 48 h. Notably, increasing cellular JMJD2B levels had no effect on p53 protein accumulation. Also, we did not observed changes in the global trimethylated H3K9 mark following JMJD2B overexpression, consistent with the assumption that JMJD2B would bind to, and therefore reorganize, only a small subset of histones in the genome.

Similarly to what we observed with 5-FU, p53 was less efficient in inducing *p21* and *PIG3* in cells treated with either doxorubicin (Figure 5C) or etoposide (Supplementary Figure S4A). On the contrary, no changes on *PUMA* and *NOXA* transcript levels were detected in these experimental settings, indicating that JMJD2B exerts selective regulation of p53 transcriptional activity by inhibiting only a subset of p53 target genes, that depends on the type of stress applied to the cell.

To unravel the functional requirement of JMJD2B for the attenuation of p53-mediated transcriptional activity, we utilized a catalytically dead variant of JMJD2B (pLPC-JMJD2B^H189A^) (Figure 5D and Supplementary Figure S4B and S4C). QPCR analysis of HCT116 p53+/+ cells stably infected with pLPC- JMJD2B^H189A^ retroviral construct showed a complete rescue in the ability of p53 to transactivate its target genes *p21* and *PIG3*, following DNA damage, when compared with cells harbouring a WT JMJD2B (pLPC-JMJD2B). These findings clearly demonstrate that the JMJD2B impingement on p53-dependent transcriptional activity is dependent on the catalytic capacity of JMJD2B.

Because JMJD2B counteracts the p53-dependent induction of key p53 targets in response to DNA damage, we wanted to assess the impact of JMJD2B silencing on *p21, PIG3* and *PUMA* gene expression (Figure 5E). QPCR analysis of HCT116 p53+/+ cells transiently transfected with small interfering RNA targeting JMJD2B (siJMJD2B) showed a 2.2-fold and 1.9-fold increased in *p21* transcripts with respect to cells transfected with a non-targeting control siRNA (siCON), in response to 5 or 10 μg/ml of 5-FU treatment for 24 h, respectively. Efficacy of siRNA delivery was tested, and *JMJD2B* gene silencing ranged from 70 to 75% in treated and untreated samples. Similarly, DNA-damage driven induction of *PIG3* mRNA was also enhanced in a statistically significant manner by knocking down JMJD2B.

### Epigenetic landscape on selected p53 target promoters following JMJD2B overexpression

**Figure 6.**
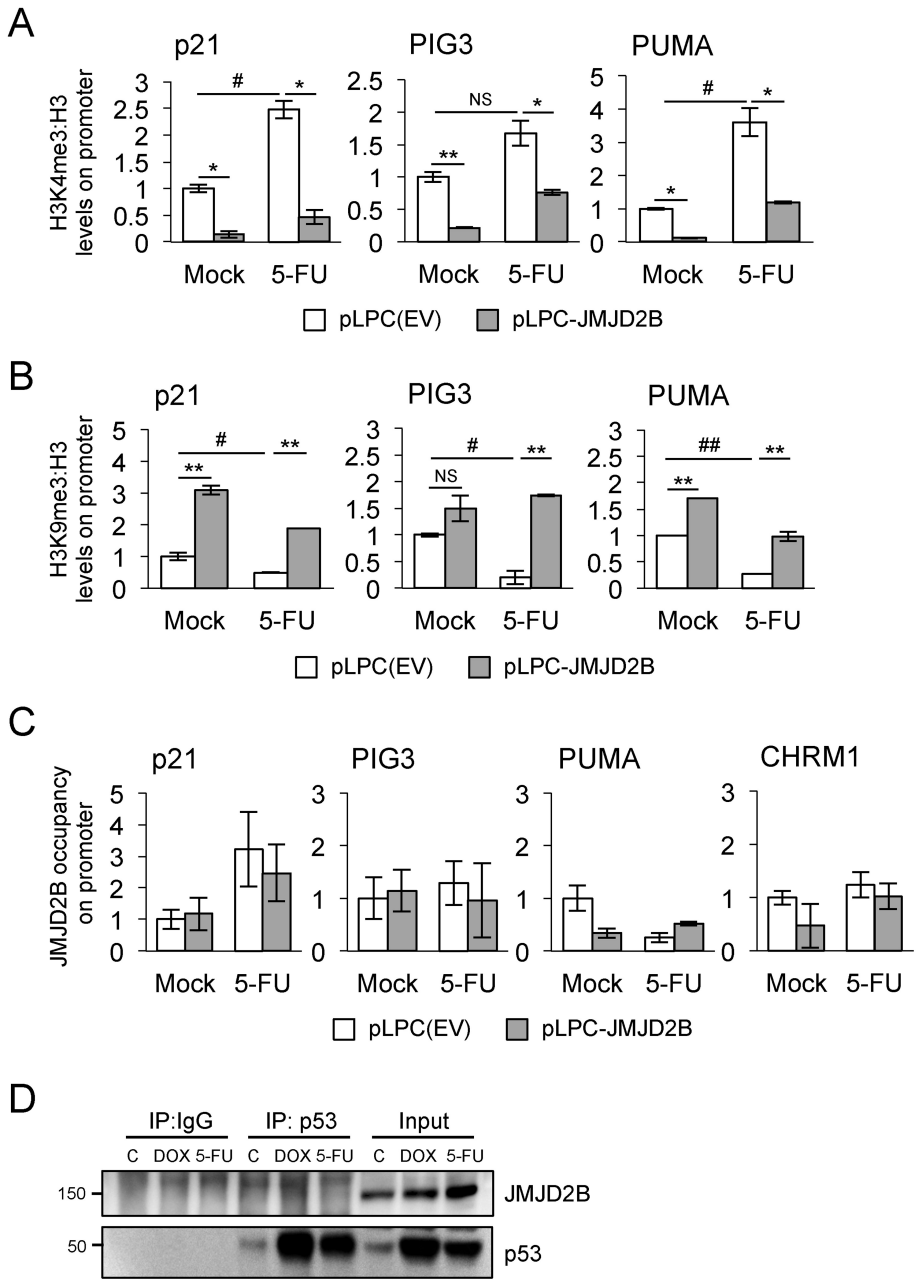
Changes in H3K4me3 and H3K9me3 marks on p53 target promoters upon JMJD2B overexpression. (**A**) H3K4me3 fold enrichment/H3 at the regions encompassing p53 consensus binding sites on *p21, PIG3 and PUMA* promoters was assessed by ChIP followed by qPCR analysis in HCT116 WT cells, stably transfected with pLPC-JMJD2B or pLPC(EV) control and treated with 5-FU for 24 h. (**B**) ChIP-qPCR analysis showing H3K9me3 levels on selected p53 target promoters in HCT116 p53+/+ cells overexpressing JMJD2B and exposed to 5-FU. Enrichments measured using a titration of pooled input samples as a standard curve, calculated as percentage of total input after subtraction of IgG signal and presented as fold change relative to pLPC(EV) untreated control (Mock), ±SEM. ∗ and # *P* < 0.05; ^∗∗^*P* < 0.01; NS, *P* > 0.05 (Student's *t*-test). (**C**) JMJD2B fold enrichment at the regions encompassing p53 consensus binding sites on the indicated promoters was assessed by ChIP-qPCR analysis. The cholinergic receptor muscarinic 1 promoter (CHRM1) was used as negative control. (**D**) JMJD2B does not coimmunoprecipitate with p53. Lysates from HCT116 WT cells were immunoprecipitated with anti-p53 (DO-1) and anti-mouse IgG antibodies and analyzed by Western blot with antibodies recognizing p53 and JMJD2B. Lysates taken before immunoprecipitation (input) were used to determine total p53 and JMJD2B levels upon doxorubicin (DOX) and 5-FU treatments.

Because H3K9 trimethylation is a hallmark of inactive chromatin, and JMJD2B specifically recognizes tri- and dimethylated lysine 9 (H3K9me3/2) on histone H3, reducing both modifications to the monomethylated state (11,12), the evidence of attenuated gene expression of *p21, PIG3* and *PUMA* seems paradoxical. In fact, JMJD2B would be expected to further activate the activity of these p53 targets rather then repressing it, by removing the inactive H3K9me3 mark. Thus, we wanted to document changes in histone methylation marks at those promoters by analysing the effect of JMJD2B overexpression on the levels of H3K4 and H3K9 trimethylation, hallmarks of active and inactive chromatin respectively. We performed ChIP followed by qPCR analysis using nuclear extracts from HCT116 WT cells, stably expressing pLPC-JMJD2B construct or pLPC-empty vector control. The ratio of H3K4me3 (Figure 6A) and H3K9me3 (Figure 6B) to bulk histone H3 on regions encompassing p53 consensus binding sites on *p21, PIG3* and *PUMA* promoters was measured in response to p53 activation. After 5-FU treatment, levels of the H3K4me3 mark were increased in a statistically significant manner for *p21* and *PUMA* promoters but not for *PIG3* (Figure 6A). Overexpression of JMJD2B abrogated this effect by reducing H3K4me3 levels, in both untreated and 5-FU treated cells, on each of the p53 target promoters analysed. In contrast, H3K9me3 levels present on *p21, PIG3* and *PUMA* promoters were significantly reduced upon p53 activation (Figure 6B), in accordance with previous findings (27). JMJD2B overexpression counteracted this effect leading to increased levels of the H3K9me3 repressive mark on p53 target promoters, both in control and stressed conditions. Together, these results provide the mechanistic link to explain that the attenuated response of *p21, PIG3* and *PUMA* expression shown after JMJD2B overexpression is reflected by a decrease in the H3K4me3 permissive mark, with an increase in the H3K9me3 repressive mark.

JMJD2B catalytic activity is required to attenuate the transcription of key p53 transcriptional targets, and increased levels of H3K9me3 are observed on the promoters of those p53 targets. However, we envision a model in which JMJD2B upregulation could enhance, by means of its demethylase activity, the expression of a transcriptional repressor, which in turn will act on a subset of p53-target genes. This hypothesis will reconcile the dependency on JMJD2B catalytic function with the evidence that JMJD2B recruitment is not required at the promoters of p53 targets. To test this hypothesis, we performed ChIP followed by qPCR analysis using nuclear extracts from HCT116 WT cells, stably expressing pLPC-JMJD2B construct or pLPC-empty vector control, and measured JMJD2B promoter occupancy on regions encompassing p53 consensus binding sites on *p21, PIG3* and *PUMA* promoters (Figure 6C). Our results show that, following 5-FU treatment, there was no significant recruitment of JMJD2B on the promoter regions of *p21, PIG3* and *PUMA* in both cell lines, thus strengthening our predicted hypothesis.

Although several lysine methyltransferases and demethylases have been identified to have critical roles in histone modification, a large body of evidence has indicated that these enzymes also regulate the methylation dynamics of non-histone proteins. Numerous studies have indeed implicated specific lysine residues within p53 as being important for its protein's transcriptional activities (1). In order to decipher whether JMJD2B directy regulates p53 protein methylation, we investigated the physical association of the putative p53/JMJD2B complex. Our immunoprecipitation studies show that JMJD2B does not form complexes with p53 in HCT116 p53 WT cells, upon treatment with the DNA damaging agents doxorubicin and 5-FU (Figure 6D). Therefore, this result excludes the possibility that a physical interaction between JMJD2B and p53 would cause the repression of p53-dependent transcriptional activation of its targets *p21, PIG3* and *PUMA*, through a direct demethylation event on p53 protein.

### JMJD2B overexpression increases tumor growth *in vivo*

Global as well as local changes in histone methylation patterns are predictive of poor prognosis and associated with patient relapse for several tumor types (38–42). In line with these observations, evidence has emerged that JMJD2B is overexpressed in numerous cancers, including breast (13–15), colorectal (16,43), gastric (44,45), prostate (17), lung and bladder malignancies (18). Also, JMJD2C, another member of the JMJD2 family, originally identified as a ‘gene amplified in squamous cell carcinoma 1’ (GASC1), is amplified and overexpressed in several tumor types, including breast cancer (46), esophageal squamous cell carcinoma (47), metastatic sarcomatoid carcinoma of the lung (48), primary mediastinal B cell lymphoma and Hodgkin lymphoma (49). In light of the data suggesting a role for JMJD2B as an oncoprotein, we wanted to elucidate whether JMJD2B overexpression is able to initiate or support tumor formation *in vivo*.

**Figure 7.**
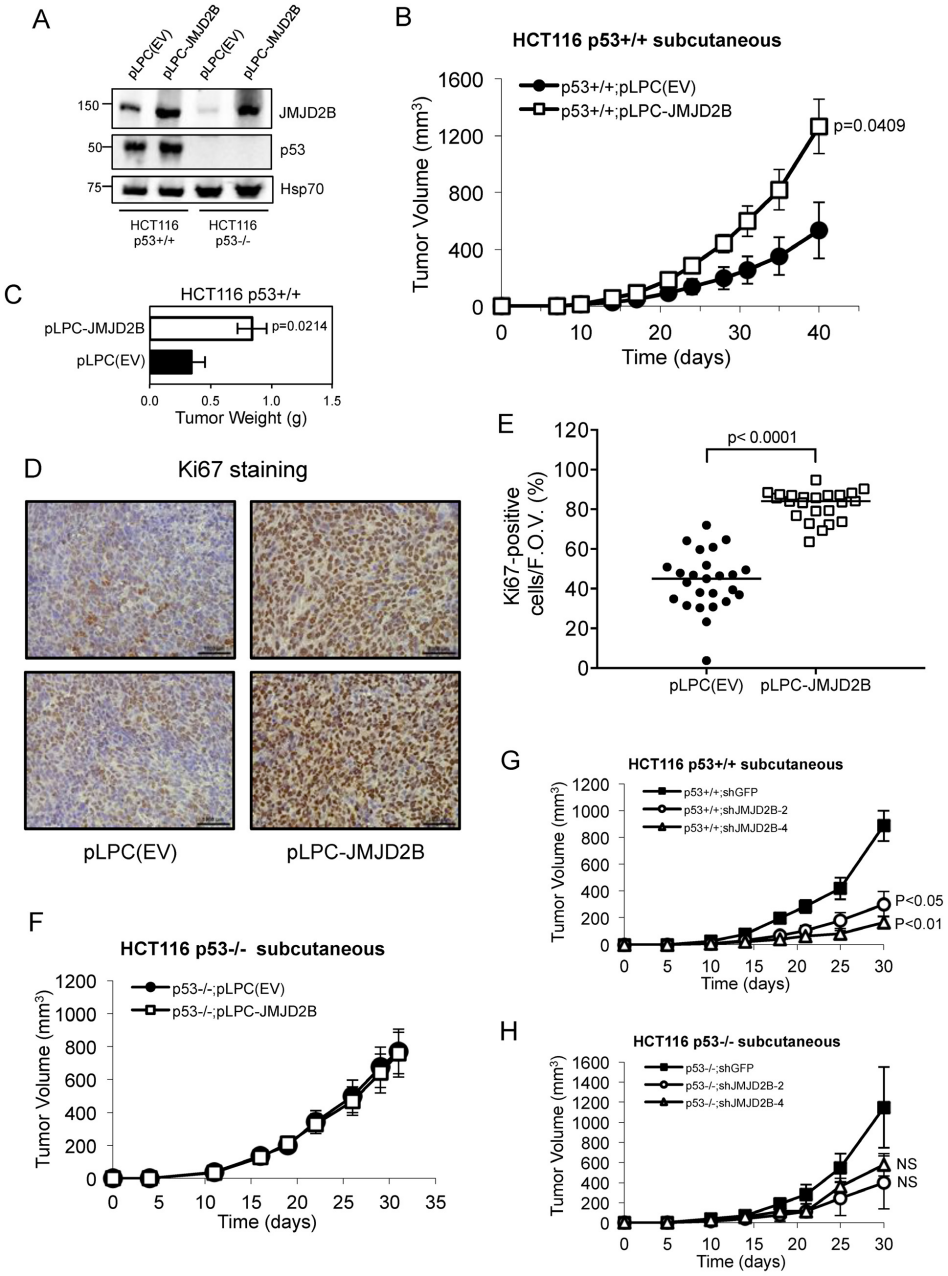
JMJD2B overexpression increases tumor burden in a xenotransplantation assay. (**A**) Western blot analysis showing p53 and JMJD2B protein levels in HCT116 p53+/+ and p53−/− cells stably transfected with JMJD2B (pLPC-JMJD2B) or control (pLPC(EV)) retroviral constructs. Hsp70 used as protein loading control. (**B**) Average tumor volume over time of xenografted HCT116 p53+/+ pLPC(EV) and pLPC-JMJD2B cells. One million cells were injected into the lower flanks of SCID hairless mice, and tumor volumes measured at the indicated intervals. Error bars represent ±SEM and significant p-value (Student's *t*-test) was calculated at the end of the experiment, compared to control (*n* = 5 per group). (**C**) Total weight of subcutaneous tumors excised from mice in (B) at day 40 following injection. Statistical significance is indicated (*P* = 0.0214). (**D**) Representative Ki67 staining by immunohistochemistry of excised tumors taken at the end of the study. Scale bar = 1000 μm. (**E**) Quantification of Ki67-positive cells per field of view (*n* = 4–6). Average percentage is represented with a horizontal bar. Significant differences were determined by a two-tailed *t*-test. (**F**) Exogenous expression of JMJD2B in HCT116 p53−/− cells does not alter their capacity to grow subcutaneous tumors *in vivo*. SCID hairless mice were injected with the indicated cell lines, and tumor volume measured at the indicated intervals (*n* = 7 per group). Error bars ± SEM. (**G** and **H**) Average tumor volumes of subcutaneous HCT116 p53+/+ (G) and p53−/− (H) tumors carrying shGFP, shJMJD2B-2 or shJMJD2B-4 lentiviral constructs, grown over a 30-day time course (*n* = 5 mice per group). Two million cells were injected into SCID hairless mice and the volume measured at the indicated intervals. Error bars ±SEM. Statistical significance is indicated compared to control. NS, *P* > 0.05 (Student's *t*-test).

Xenotransplantation of HCT116 p53+/+ cells infected with pLPC-JMJD2B-overexpressing retroviral construct (Figure 7A) into immunocompromised mice resulted in a significant increase in tumor growth relative to pPLC-empty-vector-infected cells (Figure 7B and Supplementary Figure S6A). This effect on tumorigenicity was also confirmed by measurements of total weight of subcutaneous tumors excised from mice 40-days post injection. Indeed, elevated levels of JMJD2B expression led to a greater tumor burden than the control group (Figure 7C). In addition, overexpression of JMJD2B resulted in a significant increase in Ki67 staining, a marker of proliferation, when compared with the cohort carrying pLPC(EV), confirming a putative role of JMJD2B in tumor proliferation (Figure 7D and 7E). Conversely, no significant difference was observed in subcutaneous growth (Figure 7F) and tumor weight (Supplementary Figure S6B) of pLPC(EV) and pLPC-JMJD2B HCT116 p53−/− cells, thus indicating that JMJD2B exerts a strong tumor-promoting effect in a p53-dependent manner.

To further confirm the importance of JMJD2B for tumor maintenance *in vivo*, we injected HCT116 p53+/+ cells stably transfected with either two different shRNA hairpins against JMJD2B (shJMJD2B-2 and shJMJD2B-4) or hairpin targeting a control protein (shGFP) into the dorsal flanks of SCID hairless mice (Figure 7G). Substantial JMJD2B knockdown was validated at the protein level by Western blot analysis (Supplementary Figure S6C). Both tumor volume and weight measurements taken 30-day post-injection clearly showed that genetic inhibition of JMJD2B significantly impaired *in vivo* tumor growth of HCT116 p53+/+ cells relative to control group (Figure 7G and Supplementary Figure S6E), while having a modest, not statistically significant, impact on the growth of HCT116 p53−/− cells (Figure 7H and Supplementary Figure S6F). In contrast, loss of JMJD2B in HCT116 cells cultured *in vitro* had no effect on growth rate (Supplementary Figure S6D), suggesting that the tumor microenvironment plays an important role in mediating the effects of JMJD2B on tumor growth.

Taken all together, these studies strongly indicate that JMJD2B expression exerts a strong tumor-promoting effect, which is critical for tumor initiation and maintenance of cancer cells *in vivo*, thus fulfilling its role as oncoprotein.

## DISCUSSION

The p53 tumor suppressor protein is a major sensor of cellular stresses and, upon activation, it impacts the transcription of several hundreds genes to regulate key cellular processes including cell cycle, DNA repair, apoptosis, senescence, autophagy and metabolism (1–3). Despite the ever-expanding list of new p53 target genes, our current knowledge on the role of p53 in modulating the histone code readers, writers and erasers is still limited. In the present study, we have undertaken a quantitative real-time PCR screening approach to identify which histone lysine methylating and demethylating enzymes might represent novel transcriptional targets of p53. Initially, we categorized the screened genes belonging to HDMs into three different groups, the first group being positively regulated by p53 (*JMJD2B, JARID1B*), a second group selectively repressed by p53 (*JARID2, JMJD6, LSD1, HIF1AN*) and a third group induced in response to DNA damage but in a p53-independent manner (*HAIRLESS, FBXL10, JMJD2D*). In contrast, the screening of HMTs revealed a unique pattern of regulation, as most of these genes are repressed by p53, both in stressed and unstressed conditions (*MLL1, MLL2, SETD1B, SETD1A, SETD7, SETD2, G9a, SMYD2, SUV39H1*). The identification of SUV39H1 as a p53-repressed methyltransferase in our screen is in agreement with recent reports showing that induction of p53 by various methods led to decreased RNA and protein levels of SUV39H1 (27,28), whereas concomitant exogenous expression of MDM2 and SUV39H1 cooperatively inhibited p53 activity (50). This finding is further corroborated by data showing that the p53 transcriptional target MDM2 forms a complex with SUV39H1 (50) and is able to mediate its proteosomal degradation (51).

We focused our studies on JMJD2B/KDM4B because of its paradoxical dualism, given the fact that we observed its p53-dependent activation and recent reports have described JMJD2B overexpression in numerous cancers (24,25). Furthermore, *JMJD2B* is a transcriptional target of the hypoxia-inducible factor HIF-1α, suggesting that it might aid tumors to thrive in a hypoxic environment (19,20,21). In addition, *JMJD2B* is also an estrogen receptor α target gene as well as an androgen receptor responsive gene, suggesting a role for JMJD2B in promoting hormonally responsive breast and prostate carcinogenesis, respectively (13,14,17,52).

JMJD2B is a newly identified member of the JMJD2 family, which is characterized by the catalytic Jumonji C (JmjC) domain. JMJD2B specifically targets the trimethylated lysine 9 of histone H3 (H3K9me3) for demethylation at pericentric heterochromatin and euchromatin (11). *JMJD2B* was first suggested to be a p53 target gene in TP53-depleted cells treated with 5-fluorouracil (53). A second study demonstrated that UV irradiation enhances levels of drosophila JMJD2B transcript and protein in wild-type flies, but not in p53 mutant flies (54). Recent reports suggested that p53 induction of JMJD2B after γ-irradiation causes downregulation of H3K9 trimethylation levels at the pericentric heterochromatin, thus promoting its relaxation and increasing accessibility to DNA repair factors (28).

While other members of the JMJD2/KDM4 family, JMJD2A, JMJD2C and JMJD2D are able to modulate the p53-dependent response to DNA damage, they appear to promote opposing effects on p53 transactivation. In fact, JMJD2A and JMJD2C have been recently shown to interact with p53 and be recruited at the *p21* promoter upon exposure to adriamycin (55). This increased binding of JMJD2A to the *p21* promoter leads to the inhibition of p53-mediated *p21* transcription, despite the controversial observation of a slight reduction of trimethylated H3K9 at the *p21* promoter (55). In contrast, JMJD2D is also able to bind to the *p21* promoter together with p53 upon adriamycin treatment, therefore causing a reduction in H3K9me3, but ultimately synergizing with p53 in enhancing *p21* transcription (56). Despite these past findings, it is still not known whether JMJD2 proteins demethylate p53, and no data are available regarding the transcriptional regulation exerted by p53 on the JMJD2 family, accounting for possible overlapping functions between these members and JMJD2B. In this report, we show that JMJD2A, JMJD2C and JMJD2D do not display any p53-mediated transcriptional regulation, supporting the hypothesis that, at least transcriptionally, JMJD2B is the only member of the family showing p53-responsiveness upon DNA damage.

Intriguingly, similar to the *JMJD2B* mRNA kinetics upon p53-dependent DNA damage induction, JMJD2B protein levels display patterns of a delayed induction with respect to other p53 targets such as *p21*. Physiologically, this response might represent a feedback regulation that allows p53 to repress its own transcriptional program as a means to attenuate stress inducible gene expression. During evolution, tumors overexpressing JMJD2B might have usurped this physiological pathway and consequently use it as an advantage to promote uncontrolled growth and evade p53 tumor surveillance.

Interestingly, the p53-binding site identified in our study appears to be conserved in primates but absent in the mouse or rat *JMJD2B* promoter. Notably, we were unable to detect any significant increase in *JMJD2B* expression in WT and p53−/− MEFs upon doxorubicin treatment. Thus, the mechanism by which p53 regulates JMJD2B appears not to be a conserved phenomenon in mice and humans, accounting for the possibility of tissue specificity as well as species-specific regulation.

Recently, it has been reported that a 1-kb fragment of the *JMJD2B* promoter, containing a putative p53-binding site located at −450 bp from the TSS, is able to transactivate a luciferase-base reporter construct (28), although the study did not investigate functionally a broader portion of *JMJD2B* promoter. The authors performed the experiments in a context of p53 overexpression rather then endogenous activation of p53. We identified a different functional p53-binding motif in the *JMJD2B* promoter by ChIP and promoter analysis in the endogenous setting, and we did not observe any enrichment in p53 binding on the p53 consensus site described by Zheng *et al.* (BS8 in our study). However, it is possible that this consensus site might represent an alternative and/or additional binding site for p53 to use under certain conditions.

In an effort to identify a list of key p53 target genes that were affected by JMJD2B in response to DNA damage, we show that JMJD2B is able to attenuate the response of p53 established target genes, such as *p21, PIG3* and *PUMA*, without affecting the overall cellular levels of the H3K9me3 mark, thus highlighting a repressive regulatory loop by which the p53 tumor suppressor is able to influence its own transcriptional program. In contrast, we detected no changes on *NOXA* transcript levels upon ectopic expression of JMJD2B, suggesting that JMJD2B exerts selective regulation of p53 transcriptional activity by inhibiting only a subset of p53 target genes. Because H3K9 trimethylation is a hallmark of inactive chromatin and is normally mutually exclusive with H3K4 trimethylation, which is a mark for active promoters, the recruitment of JMJD2B to active promoters would guarantee that the H3K9me3 epigenetic marks become demethylated, which will then amplify gene transcription at specific sites. Our findings of an attenuated response of *p21, PIG3* and *PUMA* expression following JMJD2B overexpression, seems paradoxical as JMJD2B would be predicted to remove the inactive epigenetic mark and facilitate gene transcription of these p53-target genes, instead of repressing their activity. Different models can be conceived to explain this regulatory feedback repressive loop exerted by JMJD2B on p53 function. One model invokes the involvement of MDM2 protein, a well know p53 gatekeeper, which can inhibit p53 activity by a variety of means. First, by binding to the transactivation domain of p53, MDM2 sterically blocks the function of that domain. Moreover, by acting as a p53-specific E3 ubiquitin ligase, MDM2 promotes the ubiquitylation and subsequent proteasomal degradation of p53 (57–59). Activation of the p53 response in cells experiencing stress involves disengagement of MDM2 and abrogation of its inhibitory effects (60). JMJD2B activation might either affect the binding affinity of MDM2 to p53, or promote a sustained increase in MDM2 levels under stress conditions, followed by ubiquitylation and, ultimately, degradation of p53. In testing this model, we were unable to detect any appreciable reduction of p53 protein levels upon JMJD2B ectopic expression in HCT116 (Figure 5B). Our data are more consistent with a direct model in which JMJD2B upregulation would enhance, by means of its demethylase activity, the expression of a transcriptional repressor, which in turn will affect a subset of p53-target genes. In testing this hypothesis, we screened for changes in the expression of known transcriptional repressors, such as *BHLHE40* (61), *ZNF420* (62) and *ID1* (63), following JMJD2B ectopic expression in HCT116 in response to DNA damage (Supplementary Figure S5). However, we could not detect any appreciable increase in the mRNA levels of *BHLHE40*, *ZNF420* and *ID1* transcriptional repressors after JMJD2B ectopic expression in HCT116. Nevertheless, consistent with a role of a transcriptional repressor, we have provided data documenting changes in histone methylation marks at the *p21*, *PIG3* and *PUMA* promoters. Specifically, we have shown a decrease of the H3K9me3 permissive mark along with an increase of the H3K9me3 repressive mark at those promoters, which recapitulates our findings of an attenuated response of these p53-targets after JMJD2B overexpression. Most importantly, we have presented data demonstrating that the JMJD2B catalytic activity is required to attenuate the p53-mediated transcription activity of *p21* and *PIG3* targets, following DNA damage. These findings, together with evidences showing that JMJD2B is not recruited on the promoters of those p53 target genes, allowed us to strengthen our model implying the requirement of a transcriptional repressor.

Alternatively, a third model might imply the ability of JMJD2B to form a complex with p53, and such physical interaction, via a demethylation process, would cause the repression of p53-dependent transcriptional activation of its targets *p21, PIG3* and *PUMA*. Indeed, lysine methylation has been shown to occur on histones as well as on non-histone proteins (64), and several studies have implicated specific lysine residues within p53 as being important for its protein's transcriptional activities (1). In particular, methylation of p53 by SET7/9 methyltransferase on K372 results in p53 stabilization and increased p21 expression (9). Conversely, the demethylase LSD1 represses p53-mediated transcriptional activation and p53-induced apoptosis by removing K370 dimethylation, which subsequently prevents the interaction between p53 and p53-binding protein 1 (TP53BP-1), a coactivator of p53 (10). However, we did not detect any interaction between p53 and JMJD2B by coimmunoprecipitation studies and, therefore, we consider it unlikely that JMJD2B is able to demethylate p53 at specific lysine residues, supporting this third hypothesis.

A final model can evoke the ability of JMJD2B to potentially demethylate H3K36me3, which would lead to decreased expression of the p53 transcriptional targets, since the removal of the trimethylated mark on H3K36 would repress transcription. In fact, comprehensive studies have determined the histone demethylase specificity of JMJD2 proteins, as well as shown that JMJD2B, similarly to JMJD2A and JMJD2C, can act on the tri- and di-methylated forms of both H3K9 and, less efficiently, H3K36 substrates (11,12,65,66). Despite the fact that much effort has gone into understanding the nature of the enzymes and their substrate specificities, little is known thus far about how JMJD2B specifically acts on H3K36me3.

Several recent studies have supported the evidence that alteration in the functioning of histone demethylases might have a profound role in cancer, given the potential contribution of an imbalance of histone methylation to oncogenic transformation (67). In line with these observations, evidence has emerged that the JMJD2/KDM4 subfamily of demethylases is highly expressed in several tumor types and, specifically, JMJD2B expression levels appears to be increased in breast, colorectal, gastric, prostate, lung and bladder malignancies (13–18,43–45). In addition, JMJD2B is required for proliferation, colony formation ability, invasion or survival of the respective cell lines (16,18,44). Similarly, it has also been reported that JMJD2B overexpression leads to H3K9 demethylation at pericentric heterochromatin that results in chromosome missegregation, supporting a role for JMJD2B overexpression in chromosomal instability, a hallmark of malignant cells (26). Nevertheless, because the association of JMJD2B overexpression with malignancy is mostly a reflection of its role in promoting cellular proliferation, caution should be used to define JMJD2B as an oncoprotein. Indeed, JMJD2B overexpression in tumor specimens might be a consequence rather than a cause of tumorigenesis. The evidence that *JMJD2B-/-* mice are viable and do not display gross abnormalities might also argue against a proposed cancer predisposition (15).

In summary, the data presented here identified the histone demethylase *JMJD2B/KDM4B* as a *bona fide* p53-responsive gene, which acts as a rheostat for the p53-dependent transcriptional program. Our findings reveal a novel auto-regulatory repressive loop by which p53, through JMJD2B activation, is able to influence its own transcriptional activity by inhibiting the expression of a subset of its target genes. More importantly, ectopic expression of JMJD2B enhances tumor growth *in vivo*, suggesting that JMJD2B may represents an attractive epigenetic target for therapeutic intervention of cancers harboring WT p53.

## Supplementary Material

Supplementary Data
