# Supplementary material for: KDM4B/JMJD2B is a p53 target gene that modulates the amplitude of p53 response after DNA damage
Source: Nucleic Acids Res. 2017 Jan 10;45(7):3674–92. doi: 10.1093/nar/gkw1281 (PMC5397198; doi:10.1093/nar/gkw1281)
Supplement: Supplementary Data [file gkw1281_Supplementary_Data.zip › nar-01610-d-2016-File003.pdf]

Supplementary Data

KDM4B/JMJD2B is a p53 target gene that modulates the amplitude of p53 response after DNA damage

Laura Castellini,[1] Eui Jung Moon,[1] Olga V. Razorenova,[2] Adam J. Krieg,[3] Rie von Eyben,[1] and Amato J. Giaccia[1,*]

[1] Department of Radiation Oncology, Stanford University School of Medicine, Stanford, CA 94305, USA
[2] Department of Molecular Biology and Biochemistry, University of California Irvine, Irvine, CA 92697, USA
[3] Department of Obstetrics and Gynecology, Oregon Health Sciences University, Portland, OR 97239, USA

Supplementary Figure 1

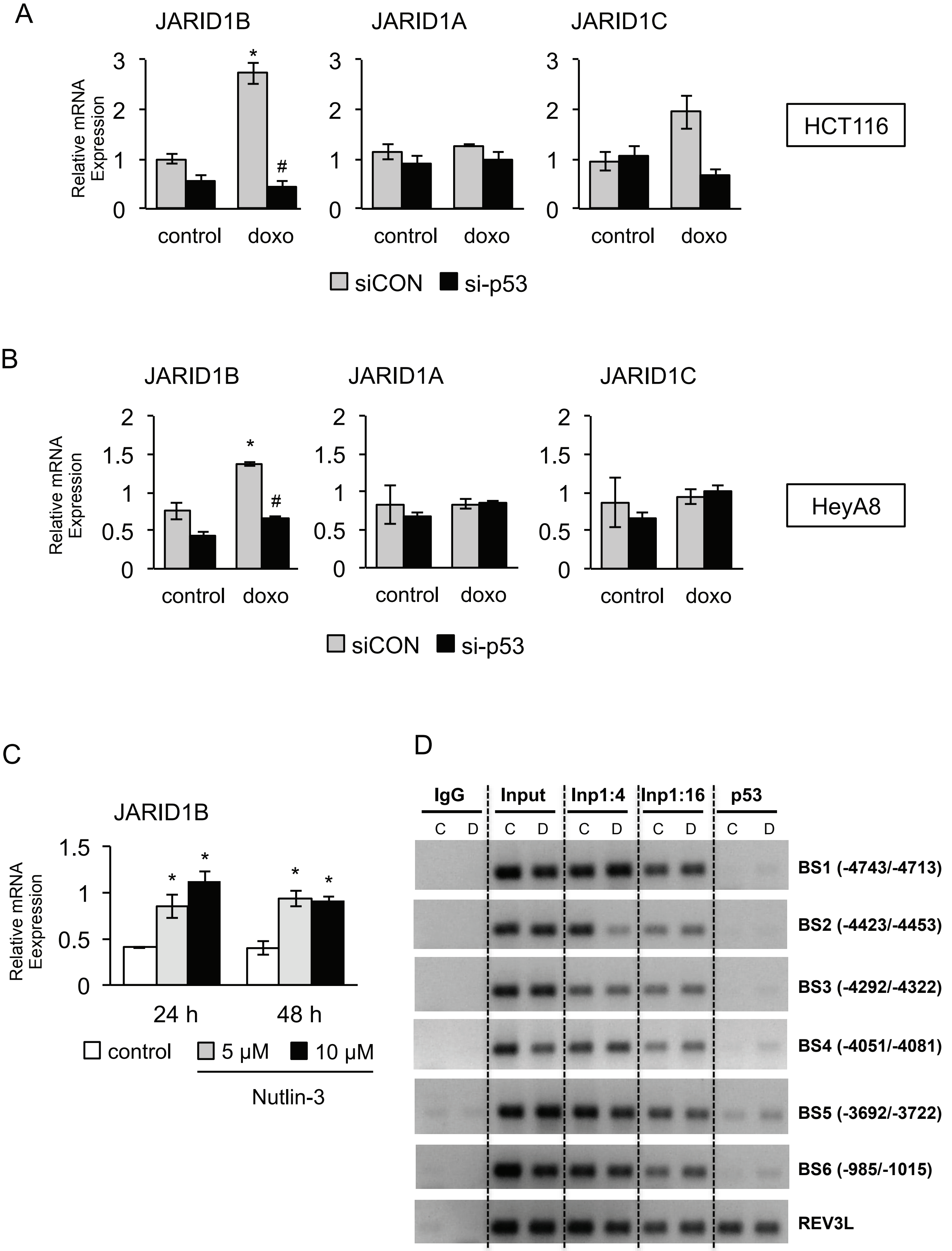

Supplementary Figure 2

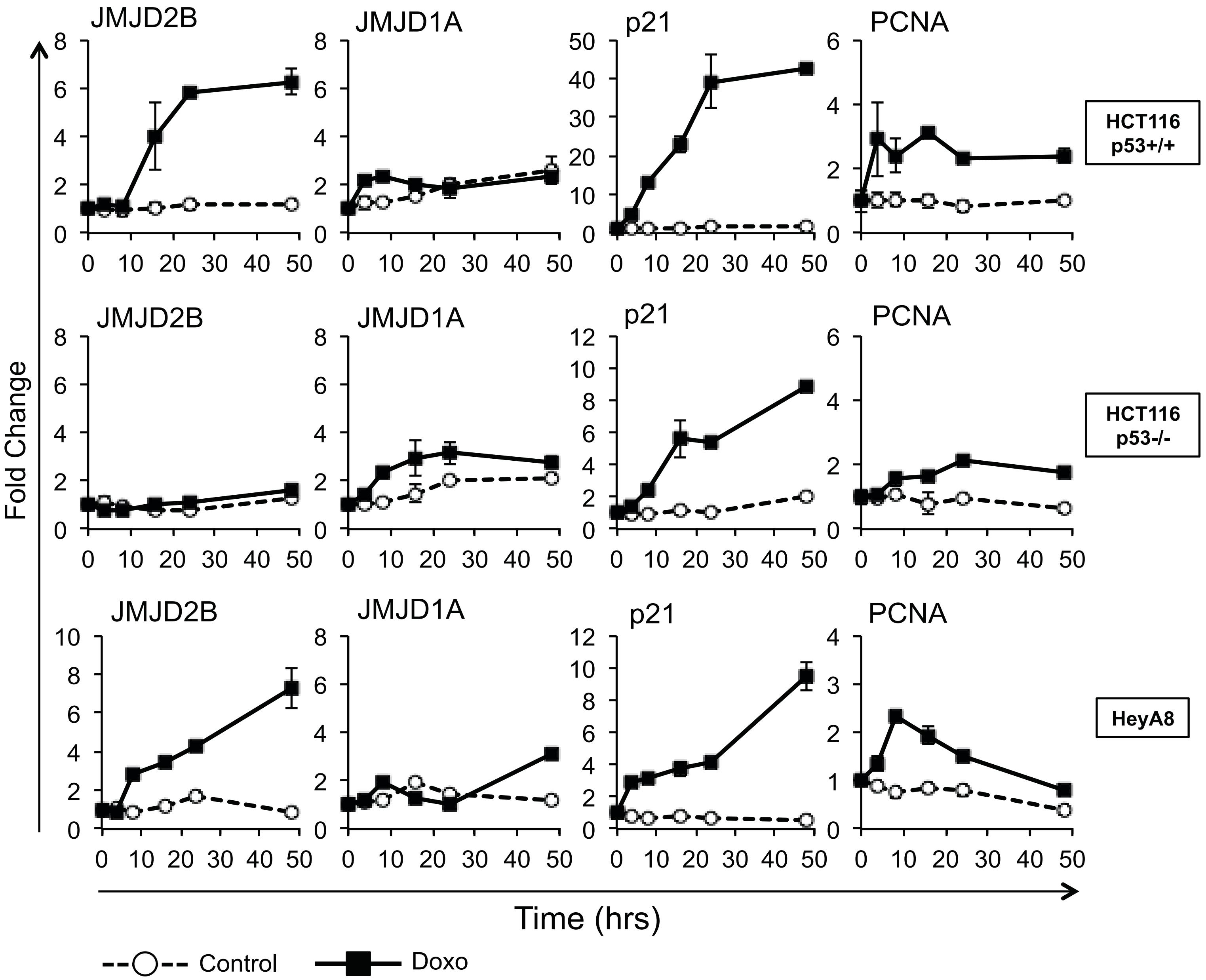

Supplementary Figure 3

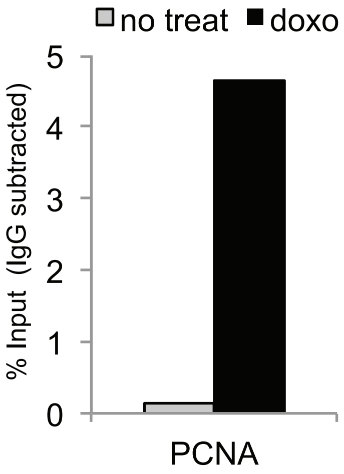

Supplementary Figure 4

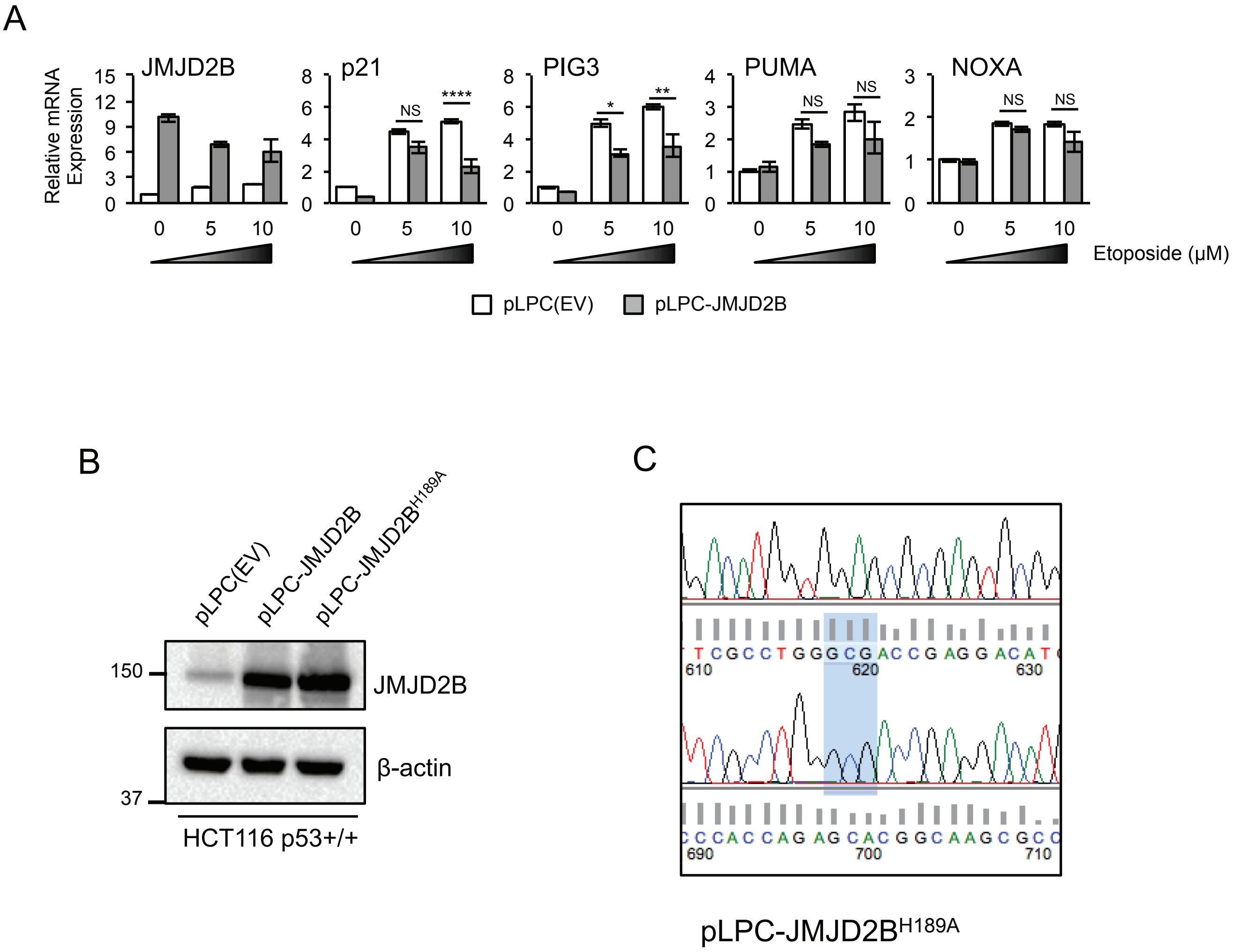

Supplementary Figure 5

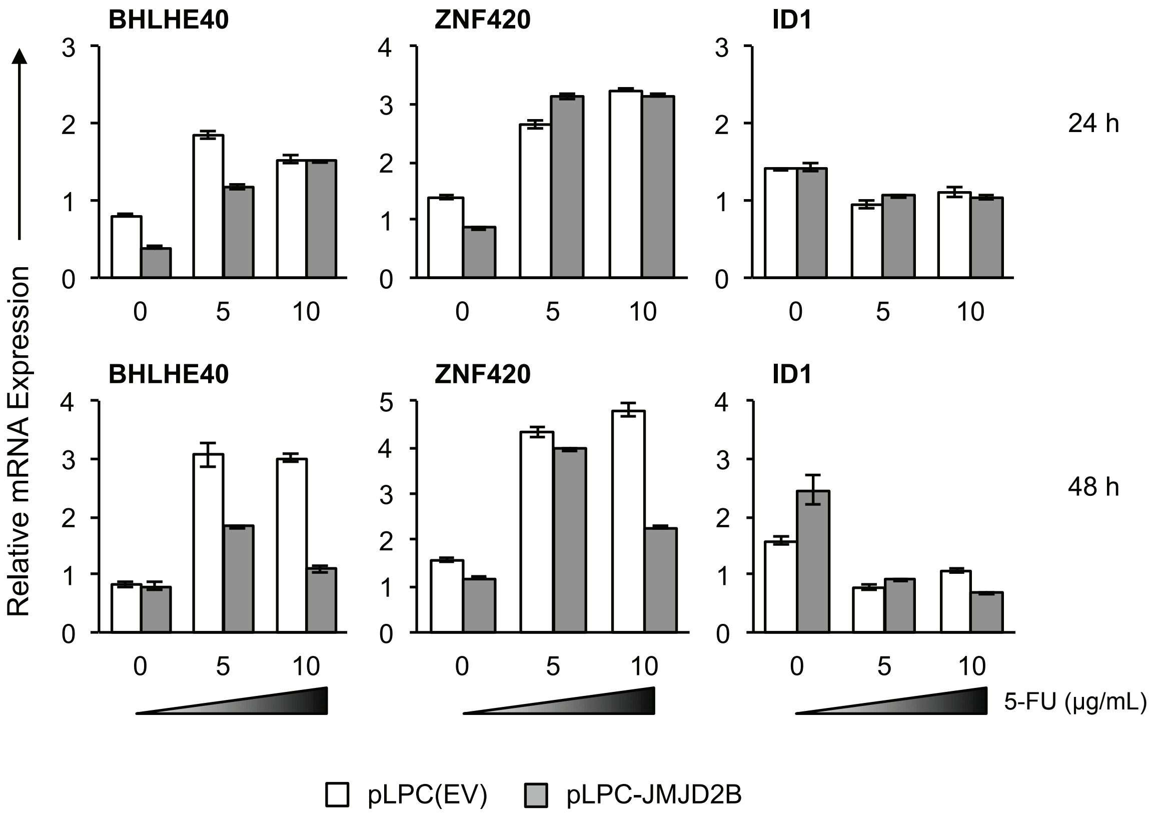

Supplementary Figure 6

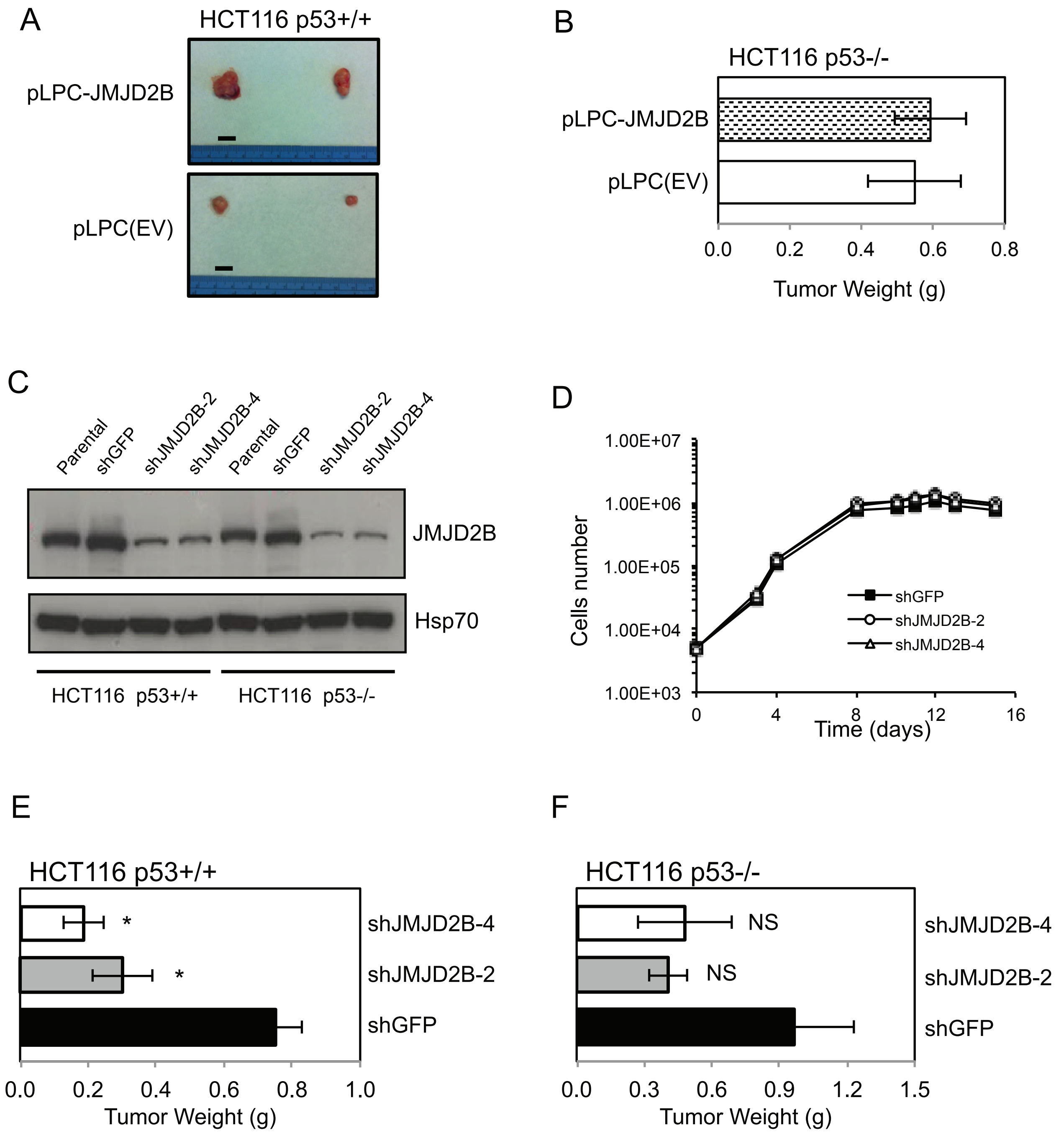

Supplementary Figure Legends

Supplementary Figure 1. JARID1B is a p53-responsive gene. Related to Figure 1. (A-B) qRT-PCR analysis of the H3K4 demethylases *JARID1A*, *JARID1B* and *JARID1C* mRNA expression in HCT116 p53+/+ (A) and HeyA8 (B) colon cancer cell lines transiently transfected with siRNAs targeting p53 (si-p53) or with a non-targeting control siRNA (siCON), and subsequently treated with 0.3 μg/mL doxorubicin (doxo) for 24 h, or left untreated (control). Data represent the average of two independent experiments, normalized to 18S, ± SEM. ∗ and #, $P<0.05$ (Student's t test). ∗, siCON control versus siCON doxo. #, siCON doxo versus si-p53 doxo. (C) *JARID1B* mRNA expression levels following treatment with 5 or 10 μM of Nutlin-3, at the indicated time points. (D) HCT116 p53 WT cells were treated 24 h with 0.3 μg/ml doxo ("D") or left untreated ("C"), and then analyzed by ChIP for p53 binding to the *JARID1B* gene promoter, using DO-1 p53 antibody. Immunoprecipitated chromatin was analyzed by PCR with primers specific to putative p53 binding sequences (BS) identified in the *JARID1B* promoter. Sequences available upon request. "Input" and "IgG" represent positive and negative controls, respectively. Two different dilution of the input were used for PCR amplification (input 1:4 and input 1:16). The REV3L promoter was used as positive control of a p53 inducible gene (1).

Supplementary Figure 2. Kinetic analysis of JMJD2B expression in response to doxorubicin. Related to Figure 2. p53+/+ and p53-/- HCT116 cells, and HeyA8 cells were treated with 0.3 μg/mL doxorubicin. RNA was harvested at 0, 4, 8, 16, 24, 48 h post treatment and analyzed by qRT-PCR. Data represent the average of two independent experiments, normalized to 18S gene expression, and plotted as fold change relative to time point zero (T=0). *p21* and *PCNA* levels were used as controls for p53-specific genes, while *JMJD1A* was used as negative control.

Supplementary Figure 3. p53 occupancy on its target promoter PCNA. Related to Figure 4. p53 binding on *PCNA* promoter, an established p53 target gene, following doxorubicin treatment for 24 h was used as positive control for ChIP assay performed in HCT116 p53+/+ cells. Same experimental conditions of JMJD2B ChIP were used.

Supplementary Figure 4. JMJD2B catalytic activity is required to modulate p53 response. Related to Figure 5. (A) *JMJD2B*, *p21*, *PIG3*, *PUMA* and *NOXA* mRNA expression in HCT116 p53 WT cells overexpressing JMJD2B or control cells (pLPC(EV)), following treatment with 5 and 10 μM etoposide. RNA was harvested 24 h post-treatment and qRT-PCR was performed. 18S gene expression used as an internal control. Error bars represent ±SEM for two independent experiments. ∗, P<0.05; ∗∗, P<0.01; ∗∗∗∗, P<0.0001; NS, P>0.05 (ANOVA). (B) Western blot analysis showing ectopic expression of WT JMJD2B (pLPC-JMJD2B) and mutant JMJD2B (pLPC-JMJD2B^{H189A}) in HCT116 p53+/+ cells versus pLPC(EV) control cells. β-actin used as protein loading control. (C) Chromatogram snapshot showing the DNA sequence traces of mutant JMJD2B (pLPC-JMJD2B^{H189A}).

Supplementary Figure 5. JMJD2B upregulation does not enhance the expression of the transcriptional repressors BHLHE40, ZNF420 and ID1, following DNA damage. Related to Figure 6. HCT116 p53 WT cells, stably transfected with pLPC-empty vector or pLPC-JMJD2B retroviral construct, were treated with 5 or 10 μg/mL of 5-FU, or left untreated. Total RNA was collected 24 h or 48 h later and subjected to qRT-PCR analysis. Expression levels of *BHLHE40*, *ZNF420* and *ID1* transcripts were determined and normalized to 18S rRNA levels. Data represent the averages from three independent experiments, each measured in triplicate. Error bars indicate ±SEM.

Supplementary Figure 6. Silencing of JMJD2B suppresses tumor growth in vivo. Related to Figure 7. (A) Representative ex-vivo tumors images of xenografted HCT116 p53+/+ cells transfected with either pLPC(EV) or pLPC-JMJD2B retroviral constructs. Scale bar = 1000 mm. (B) Total weight measurements of subcutaneous tumors excised from mice 31-days post injection of HCT116 p53-/- cells carrying pLPC-JMJD2B or pLPC(EV) control vector (n = 7 per group). (C) Western blot analysis showing downregulation of JMJD2B protein levels in HCT116 p53+/+ and p53-/- cells stably expressing shJMJD2B-1 or shJMJD2B-2 shRNA lentiviral constructs compared to untrasduced cells (parental) or control cells (shGFP). Hsp70 used as protein loading control. (D) Genetic inactivation of JMJD2B does not affect *in vitro* growth rate of HCT116 p53+/+ cells. (E-F) Total weight of subcutaneous tumors excised from mice at

day 30 post-injection of either HCT116 p53+/+ (E) or p53-/- cells (F) stably expressing shJMJD2B-1 or shJMJD2B-2 shRNA constructs compared to control (shGFP). Error bars represent ±SEM.*, $P<0.05$; “NS”, $P>0.05$ (Student’s t test).

Supplementary Tables

Supplementary Table 1. List of Human KDMs and KMTs with current Official Full Name, Official Symbol, Previous Aliases, Gene ID, Pubmed Accession Number, and Substrate Specificity, used in this study. Related to Figure 1.

KDMs					
**Official Full Name**	**Official Symbol**	**Previous aliases**	**Gene ID**	**Pubmed Accession Number**	**Substrate Specificity**
lysine (K)-specific demethylase 2B	KDM2B	FBXL10/ JHDM1B	84678	NM_032590.4; NM_001005366.1	H3K36me1/2; H3K4me3
lysine (K)-specific demethylase 2A	KDM2A	FBXL11/ JHDM1A	22992	NM_012308.2	H3K36me1/2
hypoxia inducible factor 1, alpha subunit inhibitor	HIF1AN	FIH1	55662	NM_017902.2	
hair growth associated	HR	HAIRLESS	55806	NM_005144.4	
HSPB (heat shock 27kDa) associated protein 1	HSPBAP1	HSPBAP1	79663	NM_024610.5; NM_018411.4	
lysine (K)-specific demethylase 5B	KDM5B	JARID1B/PLU-1	10765	NM_006618.3	H3K4me2/3
jumonji, AT rich interactive domain 2	JARID2	JARID2	3720	NM_004973.3	
lysine (K)-specific demethylase 7A	KDM7A	JHDM1D	80853	NM_030647.1	H3K9me1/2; H3K27me1/2
lysine (K)-specific demethylase 3B	KDM3B	JMJD1B	51780	NM_016604.3	H3K9me
jumonji domain containing 1C	JMJD1C	JMJD1C	221037	NM_032776.2	
lysine (K)-specific demethylase 4A	KDM4A	JMJD2A	9682	NM_014663.2	H3K9me2/3; H3K36me2/3
lysine (K)-specific demethylase 4B	KDM4B	JMJD2B	23030	NM_015015.2	H3K9me2/3; H3K36me2/3
lysine (K)-specific demethylase 4C	KDM4C	JMJD2C/GASC1	23081	NM_015061.3	H3K9me2/3; H3K36me2/3
lysine (K)-specific demethylase 4D	KDM4D	JMJD2D	55693	NM_018039.2	H3K9me2/3; H3K36me2/3
lysine (K)-specific demethylase 6B	KDM6B	JMJD3	23135	NM_001080424.1	H3K27me2/3
jumonji domain containing 4	JMJD4	JMJD4	65094	NM_023007.2; NM_001161465.1	
lysine (K)-specific demethylase 8	KDM8	JMJD5	79831	NM_001145348.1; NM_024773.2	H3K36me2
jumonji domain containing 6	JMJD6	JMJD6	23210	NM_001081461.1	H3R2/3
jumonji domain containing 7	JMJD7	JMJD7	100137047	NM_001114632.1	
jumonji domain containing 8	JMJD8	JMJD8	339123	NM_001005920.2	
lysine (K)-specific demethylase 1A	KDM1A	LSD1/AOF2	23028	NM_001009999.2; NM_015013.3	H3K4me1/2; H3K9me1/2

lysine (K)-specific demethylase 1B	KDM1B	LSD2/AOF1	221656	NM_153042.3	H3K4me1/2
PHD finger protein 2	PHF2	PHF2	5253	NM_005392.3	H3K9me2
PHD finger protein 8	PHF8	PHF8	23133	NM_015107.2; NM_001184897.1	H3K9me1/2; H4K20me1
lysine (K)-specific demethylase 6A	KDM6A	UTX	7403	NM_021140.3	H3K27me2/3
lysine (K)-specific demethylase 3A	KDM3A	JMJD1A/JHDM2A	55818	NM_018433.5; NM_001146688.1	H3K9me1/2
lysine (K)-specific demethylase 5A	KDM5A	JARID1A	5927	NM_001042603.2	H3K4me2/3
lysine (K)-specific demethylase 5C	KDM5C	JARID1C	8242	NM_004187.3; NM_001146702.1; NM_001282622.1	H3K4me2/3
**KTMs**					
**Official Full Name**	**Official Symbol**	**Previous aliases**	**Gene ID**	**Pubmed Accession Number**	**Substrate Specificity**
ash1 (absent, small, or homeotic)-like (Drosophila)	ASH1L	KMT2H/ASH1	55870	NM_018489.2	H3K4me1/3
DOT1-like histone H3K79 methyltransferase	DOT1L	KMT4/DOT1	84444	NM_032482.2	H3K79me1/2/3
EHMT2 euchromatic histone-lysine N-methyltransferase 2	EHMT2	G9a/KMT1C	10919	NM_001289413.1	H3K9me1/2
lysine (K)-specific methyltransferase 2A	KMT2A	MLL1	4297	NM_001197104.1; NM_005933.3	H3K4me1/2/3
lysine (K)-specific methyltransferase 2B	KMT2B	MLL2	9757	NM_014727.2	H3K4me1/2/3
lysine (K)-specific methyltransferase 2C	KMT2C	MLL3	58508	NM_170606.2	H3K4me1/2/3
lysine (K)-specific methyltransferase 2D	KMT2D	MLL4	8085	NM_003482.3	H3K4me1/2/3
lysine (K)-specific methyltransferase 2E	KMT2E	MLL5	55904	NM_018682.3; NM_182931.2	H3K4me1/2/3
nuclear receptor binding SET domain protein 1	NSD1	KMT3B	64324	NM_022455.4; NM_172349.2	H3K36me1/2
PR domain containing 2, with ZNF domain	PRDM2	KMT8	7799	NM_012231.4	H3K9me1/2/3
SET domain containing 1A	SETD1A	hSET1A/KMT2F	9739	NM_014712.2	H3K4me1/2/3
SET domain containing 1B	SETD1B	hSET1B/KMT2G	23067	NM_015048.1	H3K4me1/2/3
SET domain containing 2	SETD2	SET2/KMT3A	29072	NM_014159.6	H3K36me1/2/3
SET domain containing (lysine methyltransferase) 7	SETD7	SET7/9/KMT7	80854	NM_030648.3	H3K4me1
SET and MYND domain containing 2	SMYD2	KMT3C	56950	NM_020197.2	H3K36me2
suppressor of variegation 3-9 homolog 1 (Drosophila)	SUV39H1	KMT1A	6839	NM_001282166.1; NM_003173.3	H3K9me2/3

Supplementary Table 2. List of the p53 binding sites identified on *JMJD2B* promoter for ChIP experiments. Related to Figure 4.

**p53-canonical consensus sequence**	**TA TA<br>RRRC GYYY (0-13) RRRC GYYY<br>TA TA**
**BS1**	GGAtTTGCCT (5) GGACATtTCa
**BS2**	tGACTTGTTT (8) aGtCTTGCTC
**BS3**	tGcCATGTTg (3) AGGCTgGTCT
**BS4**	AGACcAGTCa (13) GGGCcTGCTa
**BS5**	AGcCAAGCCT (3) GGtCTTGTTT
**BS6/7**	AGACAgGTTT (1) tGcCATGTTg (3) AGGCTgGTCT
**BS8**	GcACcTGCCC (0) cAGCcTGTCC
**BS9**	GGGCAcGTgg (8) cGGCgTGTCC
**BS10**	tGctATGCCT (11) AAACTTGCCC

Sequence of the p53 binding sites (BS) identified in our study, according to the p53-canonical consensus sequence (top raw of the table). The symbols R and Y designate purine (A or G) and pyrimidine (C or T) ribonucleotides respectively. The bracketed number refers to the number of bases spacing the half sites of the consensus p53-binding site, and lowercase letters indicate deviations from the consensus. BS6/7 refers to a double p53 binding sites, because the half sites are located so close to each other that was impossible to amplify by qRT-PCR the two binding elements separately.

Supplementary Table 3. List of qRT-PCR primer sequences used in this study.

Primers for gene expression

Gene Name	Forward Primer	Reverse Primer
**KDM2B**	TGTCTGATGAGCGTGAAAGG	GTTGGAGGAATCAGCCAAAA
**KDM2A**	TGGACAAGAACTCAAGAGCAGA	GAAATTACTGTAAAGAAACCACTCCA
**HIF1AN**	TTGGGGAACCCACAAGAG	TGGACGGGATAGCAGTCAC
**HR**	GGACAGCATGATGAGCAGAA	GCATGGTATGTCCTGAAGTCC
**HSPBAP1**	GGCGACCACTCCTGTGAT	TCTCTGGCTTAAAAGGTTTGACA
**KDM5B**	AGCAGACTGGCATCTGTAAGG	GAAGTTTATCAACATCACATGCAA
**JARID2**	ATGTTCACAACGGGCATGT	CAGGTTCCTTCTCCCGTGT
**KDM7A**	GAATTACGCTCTCGAGTCTTCC	CATGTTTCTCCAGATATCTTTGTGTC
**KDM3B**	CCCCCGGTCTTCTCTACATC	GGTCAAGAAAGTTGGGTAGGC
**JMJD1C**	GAAGATTTGAAACCCAATGGAG	ACTGAACGAGGAATGCCAGT
**KDM4A**	TGGACTTGGTGGAAAAGGAG	GTCTTCAGTGTGCCAAGCAA
**KDM4B**	GGACTGACGGCAACCTCTAC	CGTCCTCAAACTCCACCTG
**KDM4C**	CGACCCTGTATACCGCACTT	CTCTCTTCCAACCCAAGCTG
**KDM4D**	GGACAAGCCTGTACCACTGAG	CTGCACCCAGAAGCCTTG
**KDM6B**	CCTTGCCCACCACTCAGTAT	CCGCCTGAGGTAGAGAACTG
**JMJD4**	AACCCCAAAGAGCACATGAC	GCACAAGTGCCAGTCTTTGA
**KDM8**	ACATCAGCATCCCCGACTAC	TACTTCCTCCCCATCACCTG
**JMJD6**	CCCAGGACGACTGTGTCAG	GCCTCCACAAGTGTCCCTAA
**JMJD7**	AAGCCCTGGGAAAGATGC	TCCTGAGACCACGCAGTAGA
**JMJD8**	GCTCCAGCTTACAGCTTTGG	AGGAACCAGCGCTTACGAC
**KDM1A**	GCCATGGTGGTAACAGGTCT	TGGCCAGTTCCATATTTACTTG
**KDM1B**	CCAGCAACTCCCCTACTGG	TGGAGTAAGCTGGATTTCCTTG
**PHF2**	CTGCTGGAGGCATTCAAAG	TTAGCTCCTTGGACTAGATGAGG
**PHF8**	GCGTGAGGAGGTTCCTGTC	GCGTCCTCTCTGGACGATAG
**KDM6A**	CATGAACACAGCACAGCAGA	ACCATGAATGAGCTTGTTGCT
**KDM3A**	TTAGCTGAACGAAAGTCACCTG	ATCTCCCAGAAAGCGAACAGA
**KDM5A**	AGCTACAAACTACCAATGGAGGA	CCCAAACTAACCAAGCATCTG
**KDM5C**	TGCTTCCATCACCAGTCAGT	GGTGGAACTTGATTTCAGAACC
**ASH1L**	TTGACTTCCAGCTTCCTTATGAT	TGGGACATCTGGCTTTTTGT
**DOT1L**	GCCACCAGACTGACCAACTC	TCGCGGTTGAGGTAGAAATC
**EHMT2**	GGACACCCCTCGTAGTGAAG	GACAGAGGCTGGAGATGAGG
**KMT2A**	AGCAGCTCTCATTTGCAGGT	ATGAGGAACACAACTGCATCA
**KMT2B**	CCAGACCTGCTGCTTGAGT	CTCAGAGCTCGAAGCCTCAC
**KMT2C**	CCACGAAAACAAAGAGGACAG	TGGGTGCTTACACTTACACAAGAT
**KMT2D**	TGCCCATGAAGGTGAAAGA	GTTTCTGTCAGCCACACACC
**KMT2E**	AAGTCCTCCAAAAATGAGCAAG	TGTGAATATTTTCCCGTGTGC
**NSD1**	CTCATCTGGGCAAAATTCAAG	GCCTCCGGTTGGAAACTT
**PRDM2**	GCTGACTTGAGTGAGAACAAGAGA	AGCGGAGGCTGTAGCTGA
**SETD1A**	CATCGAATACGTGGGTCAGA	AATGCCCTCCTGCACGTA

Gene Name	Forward Primer	Reverse Primer
**SETD1B**	GGGATCTGGACCAAAAACAA	CCCACGTAGAACTCATCGATCT
**SETD2**	TGCAGATGTGGAAGTCATACTCA	CAATATTCTAGGACAAAGGTGTTCG
**SETD7**	CCTTCACTCCAAACTGCATCT	GCAACGGTGAGCTCTTCATC
**SMYD2**	TACCAGGCCATGGGTGTC	CGTTGAGGGAGTACAAAGGATAG
**SUV39H1**	GTCATGGAGTACGTGGGAGAG	CCTGACGGTCGTAGATCTGG
**SUV39H2**	GCATTGTTTTCCACAAGAACC	TGGGCTGTGGTCAATAGAATC
**TP53 (p53)**	CCGCAGTCAGATCCTAGCG	AATCATCCATTGCTTGGGACG
**CDKN1A (p21)**	CGAAGTCAGTTCCTTGTGGAG	CATGGGTTCTGACGGACAT
**TP53I3 (PIG3)**	CAACGCTGAAATTCACCAAA	GGATCCGCCTATGCAGTCTA
**BBC3 (PUMA)**	GACCTCAACGCACAGTACGA	GAGATTGTACAGGACCCTCCA
**PMAIP1 (NOXA)**	GGAGATGCCTGGGAAGAAG	CCTGAGTTGAGTAGCACACTCG
**PCNA**	GGTGTTGGAGGCACTCAAGG	CCAAAGAGACGTGGGACGAG
**18S**	GCCCGAAGCGTTTACTTTGA	TCCATTATTCCTAGCTGCGGTATC
**BHLHE40 (DEC1)**	TGAAAGCACTAACAAACCTAATTGA	TTTCTCCCTGACAGCTCACC
**ZNF420 (APAK)**	ATGTGGGAAATCTTTTATTCGTG	TGAGTAAAGGCCATTCTACATTCTT
**ID1**	CCAGAACCGCAAGGTGAG	GGTCCCTGATGTAGTCGATGA

Primers for ChIP experiments

Promoter/p53 Binding Site	Forward Primer	Reverse Primer
**JMJD2B/p53_BS1**	CCTGCAAGCCACGAATCCT	TGACAGAAGCCAGACACAAAA
**JMJD2B p53_BS2**	TGGGCCACCTTTCTATAATCC	GATTATGCCACTGCACTCCA
**JMJD2B p53_BS3**	CTGGCCGATTTTTGTACTTTTTG	TGAGCCCGGGAGTTCGA
**JMJD2B p53_BS4**	CTCCACACCTTGCCTGTCTA	GGCTTTCAGACGTCAAGGAAC
**JMJD2B p53_BS5**	GGGTTCCTTGACGTCTGAAAG	GCAAATCTCACAAAGGACTGG
**JMJD2B p53_BS6/7**	TGAAGTGCAGTGATCTCGACTCA	GGAACACCTGAGGTCCAGAG
**JMJD2B p53_BS8**	GTCTCACGTACTCAGCCG	CACAGAGAGGAGCCAGTATTG
**JMJD2B p53_BS10**	CTCCGGTCAACCTGCTATGC	GGGGAGGGACGGAGAGACT
**p21/p53 BS**	CTGGACTGGGCACTCTTGTC	CCCTTCCTCACCTGAAAACA
**PIG3/p53 BS**	CAACGGCTCCTTTCTCTTCT	CCAGGCTTTTGGCACATTTA
**PUMA/p53 BS**	GCGAGACTGTGGCCTTGTGT	CGTTCCAGGGTCCACAAAGT
**CHRM1**	CCTTCATTGGGATCACCACG	GGAGATGAGTACCAGCAGGTTG

Supplementary Materials and Methods

Identification of putative p53 binding sites. Five kilobases upstream and one kilobase downstream of the transcriptional start site (TSS) were searched for p53 response element (p53RE) sequences using MOTIF (http://motif.genome.jp/) as described previously (1). Stringency was reduced to identify sites with a cutoff score of 75% similarity to the consensus. For ChIP-PCR, primers were designed to flank putative p53 response elements identified using Primer3 (http://frodo.wi.mit.edu/primer3/) and Primer Express Softwares (Applied Biosystems).

Supplementary References

1. Krieg, A.J., Hammond, E.M. and Giaccia, A.J. (2006) Functional analysis of p53 binding under differential stresses. *Mol Cell Biol*, **26**, 7030-7045.
